# A novel murine model of mania

**DOI:** 10.1038/s41380-023-02037-8

**Published:** 2023-03-29

**Authors:** Xinyu Li, Binjie Chen, Dianjun Zhang, Siman Wang, Yuliang Feng, Xiafang Wu, Lulu Cui, Ming Ji, Wenliang Gong, Alexei Verkhratsky, Maosheng Xia, Baoman Li

**Affiliations:** 1 Department of Forensic Analytical Toxicology, School of Forensic Medicine, China Medical University, Shenyang, China; 2 Liaoning Province Key Laboratory of Forensic Bio-evidence Sciences, Shenyang, China; 3 China Medical University Centre of Forensic Investigation, Shenyang, China; 4 Faculty of Biology, Medicine and Health, The University of Manchester, Manchester, UK; 5 Achucarro Center for Neuroscience, IKERBASQUE, 48011 Bilbao, Spain; 6 Department of Stem Cell Biology, State Research Institute Centre for Innovative Medicine, LT-01102 Vilnius, Lithuania; 7 Department of Orthopaedics, The First Hospital, China Medical University, Shenyang, China

**Keywords:** Neuroscience, Bipolar disorder, Psychology

## Abstract

Neuropathological mechanisms of manic syndrome or manic episodes in bipolar disorder remain poorly characterised, as the research progress is severely limited by the paucity of appropriate animal models. Here we developed a novel mania mice model by combining a series of chronic unpredictable rhythm disturbances (CURD), which include disruption of circadian rhythm, sleep deprivation, exposure to cone light, with subsequent interference of followed spotlight, stroboscopic illumination, high-temperature stress, noise disturbance and foot shock. Multiple behavioural and cell biology tests comparing the CURD-model with healthy controls and depressed mice were deployed to validate the model. The manic mice were also tested for the pharmacological effects of various medicinal agents used for treating mania. Finally, we compared plasma indicators of the CURD-model mice and the patients with the manic syndrome. The CURD protocol produced a phenotype replicating manic syndrome. Mice exposed to CURD presented manic behaviours similar to that observed in the amphetamine manic model. These behaviours were distinct from depressive-like behaviours recorded in mice treated with a depression-inducing protocol of chronic unpredictable mild restraint (CUMR). Functional and molecular indicators in the CURD mania model showed multiple similarities with patients with manic syndrome. Treatment with LiCl and valproic acid resulted in behavioural improvements and recovery of molecular indicators. A novel manic mice model induced by environmental stressors and free from genetic or pharmacological interventions is a valuable tool for research into pathological mechanisms of mania.

## Introduction

Neuropathological mechanisms of manic syndrome or manic episodes in bipolar disorder are poorly characterised, as the research progress is severely limited by the paucity of appropriate animal models [1]. At present, the most commonly used murine mania models are based either on pharmacological treatments [2] or genetic modifications [3, 4]. Another mouse model of mania was established through sleep deprivation [5, 6]. However, none of these models faithfully reproduces the pathology. Treatments with amphetamine or cocaine interfere with the analysis of the pharmacology of antimanic drugs and recruit pathological mechanisms of drug abuse. Genetically modified animal models are based on manipulations with the suspected risk genes of mania or bipolar disorder (BD) that do not cover all clinical presentations, while models built through sleep deprivation display multiple and diverse outcomes, including cognitive impairments or major depression [7, 8]. Previous attempts to establish manic models ignored many environmental factors (such as circadian and seasonal disturbance) that are known to contribute to the aetiology of mania [9].

In this study, we developed a novel mice model of mania by exposing animals to a series of chronic unpredictable rhythm disturbances (CURD), which include disruption of circadian rhythm, sleep deprivation, exposure to cone light, with subsequent interference of followed spotlight, stroboscopic illumination, high-temperature stress, noise disturbance and foot shock. To validate this model, we compared biological and behavioural indicators measured from CURD-treated mice, healthy mice and mice subjected to a depression-inducing protocol of chronic unpredictable mild restraint (CUMR). Our novel model of mania reproduces major pathological characteristics of the manic syndrome and demonstrates sensitivity to commonly employed antimanic pharmacological treatments.

## Materials and methods

### Materials

Most chemicals, reagents and antibodies were purchased from Invitrogen (CA, USA) and Thermo Fisher Scientific (Waltham, MA, USA). The details of materials and methods are described in Supplementary Data 1.

### Animals

Male wild-type C57BL/6 mice (#000664) and B6-Tg-Aldh1l1-EGFP mice (#030247) aged 10–12 weeks were purchased from the Jackson Laboratory (Bar Harbor, ME, USA). All experimental protocols were approved by the Institutional Animal Care and Use Committee of China Medical University, No. [2020]102.

### Chronic unpredictable rhythm disturbance (CURD) regimen

Male mice were exposed to the pattern of stressors for 3 weeks. The normal rhythm was alternated for 12 h between light and darkness. The circadian rhythm was interfered with by shortening the darkness time (mode 1) or the light time (mode 2) by 6 h, sleep deprivation (6 h), stroboscopic illumination in the dark (120/min for 12 h), irregular exposure to a solid cone light during the dark period (12 h), a spotlight constantly following a mouse during darkness period (12 h), an increase of environmental temperature to 40–45 °C (30 min), noise (100 dB for 12 h), and foot shock (0.8 mA for 2 s). Two of these eight stressors were randomly selected and applied once every day. The detailed everyday treatment protocol is shown in Supplementary Table 1.

### Chronic unpredictable mild restraint (CUMR) regimen

Male mice were randomly exposed to the following stressors for 3 weeks: restricting activity (4 h), damp bedding (12 h), cage shaking (40/min for 10 min), tail suspension (10 min), forced swimming (10 min 25 °C), 45° tilting cage (12 h). Two of these six treatments were randomly scheduled once every day, and continued for 3 weeks; a detailed everyday treatment protocol is shown in Supplementary Table 1.

### Ethogram

As reported previously [10, 11], mice status was evaluated according to general appearance parameters (GAP) assessments.

### Sucrose preference test

The sucrose preference is a reward-based test and a measure of anhedonia. In brief, mice were provided with two pre-weighed bottles, one bottle containing 2.5% sucrose water solution and another filled with plain water, for 12 h. The total intake volume of pure and sucrose water was calculated separately [12–14].

### Sucrose pellets preference test

The sucrose pellets preference test is a reward-based test and a measure of anhedonia. The white and yellow pellets were supplemented with tasteless food colouring, 1 g of sucrose was randomly added to the white and yellow pellets. Mice were provided with sweet and unsweetened pre-weighed pellets for 6 h. The total weight of every colour pellet remaining at the end of 6 h was recorded.

### Tail suspension test (TST)

The tail suspension is a behavioural despair-based test. The duration of immobility in the last 4 min was calculated by the Labstate software (YHTSM, Wuhan Yihong Technology Co. Ltd, China) [8, 15].

### Forced swimming test (FST)

The test was made as described previously [16]; the time of immobility was recorded during 4 min period followed by 2 min of habituation.

### Open field test

The open field test evaluates the autonomous activity and exploration behaviours and anxiety of experimental animals in a new environment. Mice were placed in the open field box (50 cm × 50 cm × 50 cm), the central area covering one-third of the total open field area and behaviour was recorded for 5 min [17].

### Three-chambered sociability test

Three-chambered sociability test measures the social exploration of mice. The three-chamber (empty, central, and stranger) device is a Perspex box (60 cm × 40 cm). Movements of mice and time spent in each chamber and in social circles were recorded by a video-tracking system Labstate [18].

### Pentobarbital-induced sleep test

The pentobarbital-induced sleep test is a common method for evaluating sleep in mice. The test was set to take place between 1:00 p.m. and 5:00 p.m. Each test mouse was intraperitoneally injected with pentobarbital sodium (50 mg/kg). The sleep latency (the time spent between the injection of pentobarbital sodium and the disappearance of the righting reflex) and sleep duration (the time spent between the mice asleep and the appearance of the righting reflex) were recorded [19].

### Total sleep time calculation

In order to explore the sleep duration, we conducted a 24 h video recording, from which the sleep duration was measured.

### Morris water maze test

After the consecutive 5-day trials, the mice were given 60 s to explore, and the time spent in the target quadrant was collected for each mouse [12, 14].

### Rotating rod test

The rotating rod test assesses the balancing ability as a proxy for motor control by recording time spent on the rotating rod. Each mouse was placed on a rotating bar, and the latency before falling was recorded with a maximum of 90 s [12, 14, 17].

### Pole test

Each mouse was paced head-upward on the top of a vertical rough-surfaced pole; the turn downward from the top of the pole (T-turn time) and the descent to the floor (T-LA) time was recorded [12].

### Bite ability test

The bite ability test was used to evaluate the bite force of mice, which could indirectly reflect irritability and physical strength. Mice were given an apple tree branch of the same shape, size and weight between 1:00 a.m. and 3:00 a.m. The weight of the torn debris was measured.

### Assessment of the glymphatic system

The fluorescence tracers (OA555, FITC-D3) were reconstituted in artificial cerebrospinal fluid (ACSF) at a concentration of 0.5%. The tracers were infused into the subarachnoid CSF through cisterna magna; 30 min after the start of the infusion, the brain tissue was collected, cut into 50 μm slices and imaged using AxioScan microscope (Carl Zeiss, Jena, Germany) [12].

### Pharmacological treatment

LiCl was intraperitoneally injected at a dose of 45 mg/kg/day; valproic acid (VPA) was intraperitoneally injected at a dose of 120 mg/kg/day in the last 3 weeks of the CURD regimen.

### Western blotting

After electrophoretic separation, the gels were transferred to polyvinylidene fluoride (PVDF) membranes; the samples were blocked by skimmed milk powder; membranes were incubated with the primary and secondary antibodies. Gels were visualised and analysed as described previously [20].

### Immunofluorescence (IF)

Brain slices were permeabilised by incubation with donkey serum. Slices were incubated with primary and secondary antibodies separately. Nuclei were stained with 4′, 6′-diamidino-2-phenylindole (DAPI). Images were captured using a confocal scanning microscope (DMi8, Leica, Wetzlar, Germany) [21].

### Microdialysis and HPLC-MS analysis

A guide cannula (CMA 7) was implanted into the right prefrontal cortex. Artificial cerebrospinal fluid (ACSF) was perfused through the microdialysis probe at 1 μL/min, with samples collected 3 h after probe insertion. The interstitial fluid 5-HT levels were measured immediately [8] with high-performance liquid chromatography (HPLC; Agilent 1260 Infinity LC system, USA) in tandem with a mass spectrometry (MS) system [22]. The quantification was performed using peak area ratios from calibration standard curves and was normalised to the total protein levels in the samples as determined by the Lowry method.

### Fluorescence-activated cell sorter (FACS)

B6-Tg-Aldh1l1-EGFP mice were used for isolating astrocytes. A cell suspension from the cortex was prepared as previously described [17, 23]. The cortices from three mice were pooled into one sample. The labelled cells were sorted and collected using the BD FACSAria Cell Sorting System (San Jose, CA) [17, 24]. The purity of sorted astrocytes was ascertained by detecting mRNA of the cell-specific marker as described previously [13, 23, 25, 26].

### Clinical sample collection and preparation

This study was authorised and approved by the Medical Ethics Committee of China Medical University (No. [2022]452, No. [2023]58) and registered on the Chinese Clinical Trial Registry (registration number: ChiCTR2200061158), and the informed consent was obtained from all subjects. Blood samples were collected from 30 healthy subjects, 30 patients with major depressive disorder (MDD) patients and 30 patients with bipolar disorder (BD), diagnosed in the First Hospital of China Medical University. Both manic and depressive symptoms of patients were estimated by the Clinician-Administered Rating Scale for Mania (CARS-M) [27] and the Hamilton Depression Rating Scale (HDRS) [28], respectively. The demographics of healthy subjects and patients are shown in Supplementary Table 2.

### Arachidonic acid (AA) and prostaglandin E2 (PGE2) assays

Standard curves were made by the measured optical densities of the standard solutions to calculate the concentrations of AA and PGE2 in the samples; the results were normalised by the protein content.

### Statistical analysis

The animal tests and clinical studies were performed by an investigator blinded to the experimental conditions. Power Analysis and Sample Size (PASS) 2020 software (NCSS, LLC, Utah, USA) was used to estimate the suitable sample number in clinical and animal experiments. One-way analysis of variance (ANOVA) followed by a Tukey’s or Dunnett’s post hoc multiple comparison test for unequal replications were performed by GraphPad Prism 5 software (GraphPad Software Inc., La Jolla, CA, USA) and SPSS 24 software (International Business Machines Corp., NY, USA). All data are presented as the mean ± SD, and the value of significance was set at *p* < 0.05. For correlation analysis, Spearman correlation analysis on the data, analysis and visualisation was performed using R 3.6.3 and Empower (R) software (X&Y Solutions, Inc., MA, USA), and operated R package ggplot2 to do visualisation.

## Results

### Establishment of mania and depression models

We used a series of environmental interventions to establish a novel murine model of mania. We defined the complex of these interventions as CURD, which subjected animals to a regimen of eight stressors applied for three weeks as detailed in the methods (Supplementary Fig. 1a–i).

We also developed a protocol to induce depressive-like behaviours by employing the set of restrain-related stressors. We defined this regimen as CUMR, which is based on and further advances the widely used chronic unpredictable mild stress (CUMS) protocol [13, 14]. A major difference between CUMR and CUMS is the exclusion of rhythm disturbances such as food deprivation, group housing and stroboscopic illumination. The CUMR stressors are summarised in Supplementary Fig. 1k–p.

### Behavioural testing

**Fig. 1:**
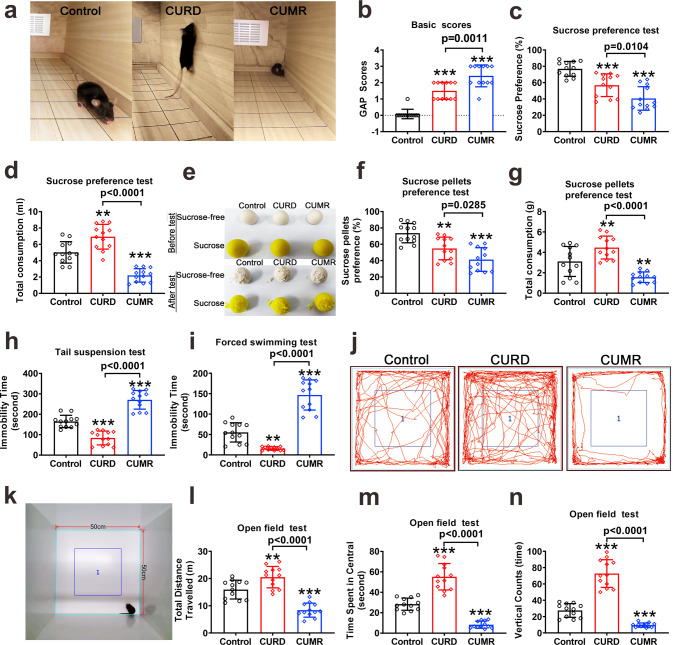
Anhedonia and anxiety behavioural measures of CURD and CUMR mice. After treatments with sham, CURD or CUMR for 3 weeks, the mice behaviours in three groups were tested. **a** The representative pictures of mice in control, CURD and CUMR groups. **b** The general appearance parameters (GAP) scores were used to assess the ethogram. **c** The percentage of sucrose preference was used to test anhedonia. **d** The total intake volume of water used to test appetite. **e** The representative pictures of pellets in the sucrose pellets preference test. **f** The percentage of sucrose pellets preference was operated to assess anhedonia. **g** The total intake weight of pellets was used to assess appetite. The immobility time in tail suspension (**h**) and forced swimming tests (**i**) were recorded in the last 4 of 6 min. **j**–**n** In an open field test, the picture of the open field was shown in **k**. The representative trails of mice treated in control, CURD and CUMR groups were shown in **j**. The total travelled distance (**l**), the time spent in central space (**m**) and the times of vertical counts (**n**) were recorded in 5 min. Data were presented as mean ± SD, *n* = 12 per group. One-way ANOVA for comparisons including more than two groups; unpaired two-tailed *t*-test for two group comparisons. Significant different from control group: **p < 0.01 and ***p < 0.001.

Exposure to CURD and CUMR regimens produced two distinct phenotypes. Most overt was the general appearance of animals: mice subjected to CURD were hyperactive and restless, whereas mice subjected to CUMR were passive and idle (Fig. 1a and Supplementary Videos 1–3). General appearance parameters (GAP) scores were used to obtain the ethogram (Fig. 1b). The GAP scores increased significantly in CURD (*p* < 0.0001, *n* = 12) and were even higher in CUMR groups compared to CURD. Sucrose uptake in CURD and CUMR groups decreased significantly (Fig. 1c) to 73.97 ± 18.09% and 52.89 ± 12.75% of the control group (*p* < 0.0001, *n* = 12). The total intake volume of water in the CURD group increased to 138.21 ± 30.20% of the control group (*p* = 0.0032, *n* = 12; Fig. 1d), whereas in the CUMR group, it decreased to 43.85 ± 16.37% of the control group (*p* < 0.0001, *n* = 12; Fig. 1d). To further assess consumption of solid food, we used a sucrose pellets preference test (Fig. 1e–g). The intake percentage of sucrose sweet pellets decreased in both CURD and CUMR groups, being, respectively, 74.51 ± 18.79% (*p* = 0.0017, *n* = 12) and 56.12 ± 19.63% (*p* < 0.0001, *n* = 12) of control (Fig. 1e, f). The total intake weight of pellets in the CURD group increased to 143.80 ± 18.79% of the control group (*p* = 0.0181, *n* = 12; Fig. 1g), while in the CUMR group, it decreased to 50.28 ± 16.74% of the control (*p* = 0.0023, *n* = 12; Fig. 1g). Immobility times in despair-related tail suspension and forced swimming tests in CURD group were significantly decreased (*p* < 0.0001, *n* = 12); to the contrary, the immobility time in CUMR group increased (*p* < 0.0001, *n* = 12) (Fig. 1h, i).

The recorded total travel distance (anxiety-related open field test, Fig. 1j–l) in the CURD group increased significantly to 128.74 ± 24.66% of the control (*p* = 0.0061, *n* = 12). In contrast, the travel distance in the CUMR group decreased to 52.71 ± 16.16% of control values (*p* < 0.0001, *n* = 12). Time spent in the central area, as well as times of vertical counts, were increased in CURD group, but reduced in CUMR group (Fig. 1m, n; *p* < 0.0001, *n* = 12).

**Fig. 2:**
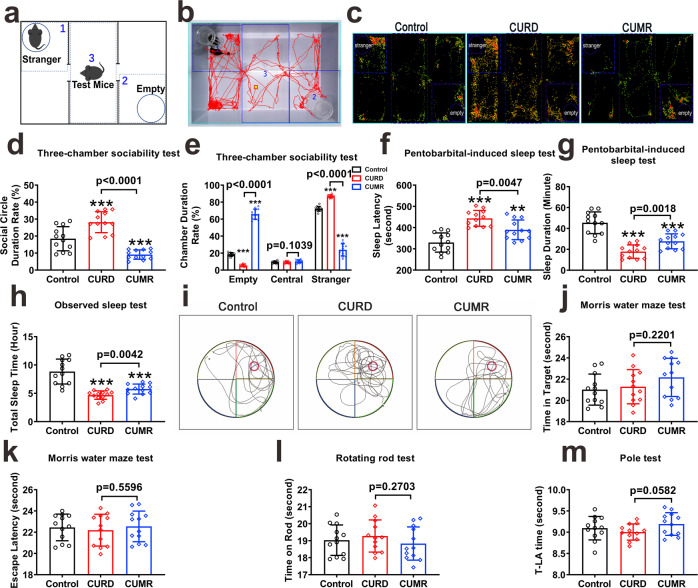
The social and cognitive behavioural measures of CURD and CUMR mice. After treatments with sham, CURD or CUMR for 3 weeks, mice behaviours were tested. **a**–**e** Schematic diagram of three-chamber test and the representative trail of CURD-treated mice are shown in **a** and **b**. The representative hot points images of the mice in control, CURD and CUMR groups (**c**), the ratio of social circle duration (**d**) and chamber duration (**e**) recorded for 10 min. After intraperitoneal injection of pentobarbital at 50 mg/kg dose, the time of sleep latency (**f**) and sleep duration (**g**) were recorded. The total sleep time of mice without interference were recorded in 24 h (**h**). **i**–**k** In Morris water maze test, the representative trails of mice in the control, CURD and CUMR groups are shown in **I**, the time in the target quadrant (**j**) and the time of escape latency (**k**) were recorded in 60 s. The time on the rotating rod (**l**) and the T-LA time in pole test (**m**) were recorded for control, CURD and CUMR groups. Data were presented as mean ± SD, *n* = 12 per group. One-way ANOVA for comparisons including more than two groups; unpaired two-tailed *t*-test for two group comparisons. Significant different from control group: **p < 0.01 and ***p < 0.001.

In the three-chambered sociability test (Fig. 2a, b), the trail of the test mouse was recorded for 10 min, and the representative hot points images of the test mouse in three chambers were detected for control, CUMR and CURD groups (Fig. 2c). Social duration ratio in CURD mice with the stranger mice increased to 152.69 ± 33.42% of the control group (*p* = 0.0017, *n* = 12), while the social duration ratio of CUMR mice decreased to 49.58 ± 14.92% of the control group (*p* = 0.0003, *n* = 12, Fig. 2d). As compared with the control group, the time spent in the empty chamber significantly decreased in CURD group, while being increased in CUMR group. The time spent in the stranger chamber increased in the CURD group and decreased in the CUMR group. The residence time in the central chamber was the same for all groups (Fig. 2e). In the pentobarbital-induced sleep induction test, the sleep latency times were prolonged in both CURD and CUMR mice (Fig. 2f). In contrast, the sleep duration time of CURD and CUMR groups was significantly reduced (*p* < 0.0001, *n* = 12; Fig. 2g). The total sleep time of CURD and CUMR mice was also less than in control (Fig. 2h). The total sleep time of CURD group was shorter than CUMR-treated mice (*p* = 0.0042, *n* = 12; Fig. 2h). In Morris water maze test (Fig. 2i), the time spent in the target quadrant and the time of escape latency were similar in all groups (Fig. 2j, k). Likewise, the motor function assessed by rotating rod and pole tests was not affected. Neither time kept on the rod nor the T-LA time differed between groups (Fig. 2l, m).

### Behavioural comparison of mania model with pharmacological models and efficacy of specific pharmacological treatment

**Fig. 3:**
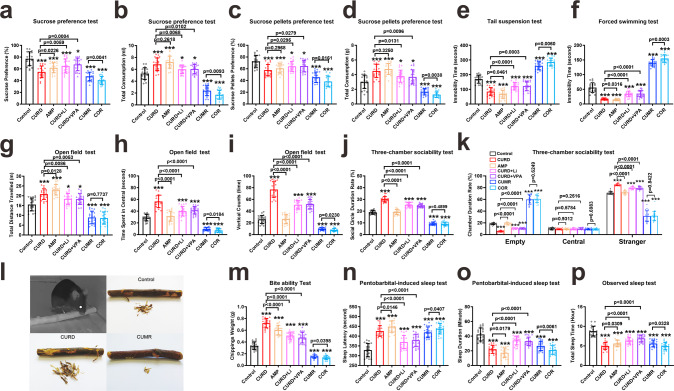
Behavioural indicators for CURD and CUMR mice treated with specific drugs as compared with amphetamine and corticosterone models. **a** The percentage of sucrose preference. **b** The total intake volume of water used to test appetite. **c** The percentage of sucrose pellets preference was operated to assess anhedonia. **d** The total intake weight of pellets was used to assess appetite. The immobility time in tail suspension (**e**) and forced swimming tests were recorded in the last 4 min of 6 min. **f** In the open field tests, the total travelled distance (**g**), the time spent in central space (**h**) and the times of vertical counts (**i**) were recorded in 5 min. In the three-chambered sociability test, the ratio of social circle duration (**j**) and chamber duration (**k**) were recorded in 10 min. **l** The picture mice of gnawing in the darkness and the representative images of stick scrap gnawed by mice in control, CUMR and CURD groups. **m** The chippings weights gnawed by mice in 2 h. After intraperitoneal injection of pentobarbital at 50 mg/kg dose, the time of sleep latency (**n**) and sleep duration (**o**) were recorded. The total sleep time of mice without interference were recorded in 24 h (**p**). Data were presented as mean ± SD, *n* = 20 per group. One-way ANOVA for comparisons including more than two groups; unpaired two-tailed *t*-test for two group comparisons. Significant different from control group: *p < 0.05, and ***p < 0.001.

We further compared the behavioural performance of mice exposed to CURD or CUMR regimen with pharmacological manic and depressed mice models and also assessed the efficacy of specific pharmacological treatments with Li^+^ and VPA (Fig. 3). The amphetamine (AMP) model [29] was established by intraperitoneal injection of 2.5 mg/kg amphetamine for 10 days. For the corticosterone (COR) model, 20 mg/kg corticosterone was intraperitoneally injected for 28 days [15, 30].

Sucrose uptake significantly decreased in CURD, AMP, CURD + Li^+^, CURD + VPA, CUMR and COR groups (*p* < 0.0001, *n* = 20; Fig. 3a). The sucrose intake in the CURD group was lower than in AMP group (*p* = 0.0226, *n* = 20; Fig. 3a). Administration of Li^+^ or VPA in CURD groups recovered sucrose intake (*p* = 0.0059, *p* = 0.0004, *n* = 20; Fig. 3a), while it remained higher in CUMR group when compared with COR group (*p* = 0.0041, *n* = 20; Fig. 3a). The total intake of water (Fig. 3b), of CURD and AMP groups, significantly increased (*p* < 0.0001, *n* = 20) when compared with the control group, whereas in CUMR and COR groups water consumption decreased (*p* < 0.0001, *n* = 20). Treatment of CURD mice with either Li^+^ or VPA significantly reduced the water intake (*p* = 0.0068, *p* = 0.0102, *n* = 20; Fig. 3b). Similar trends were obtained for the intake of solid sweet sucrose pellets (Fig. 3c, d). In the despair-related tail suspension and forced swimming tests (Fig. 3e, f), the immobility time in CURD and AMP groups decreased significantly as compared with the control group (*p* < 0.0001, *n* = 20). Treatment of CURD mice with Li^+^ or VPA, significantly increased the immobility time (Fig. 3e, f). However, in these two despair-related tests, the immobility time in both CUMR and COR groups was increased when compared with the control group (*p* < 0.0001, *n* = 20; Fig. 3e, f).

Total travelled distance (Fig. 3g–i) significantly increased in CURD and AMP groups (*p* < 0.0001, *n* = 20), while decreasing in CUMR and COR groups (*p* < 0.0001, *n* = 20). In the open field test, the time recorded in the central region increased only in the CURD group (*p* < 0.0001, *n* = 20), with no significant difference between the control and AMP groups. The time in the central region was reduced in CUMR and COR mice (*p* < 0.0001, *n* = 20; Fig. 3h). Calculated vertical counts in the CURD group was higher than in the control group (*p* < 0.0001, *n* = 20), whereas the vertical times were reduced in CUMR and COR mice as compared with the control group (*p* < 0.0001, *n* = 20) with no significant difference between control and AMP group (Fig. 3i). Treatment of CURD-exposed mice with Li^+^ and VPA significantly decreased the total distance, the time spent in the central region and the vertical counts (Fig. 3g–i). Social circle duration ratio was significantly increased in CURD-treated mice (*p* < 0.0001, *n* = 20), while decreasing in CUMR and COR-treated animals (*p* < 0.0001, *n* = 20; Fig. 3j). Administration of Li^+^ and VPA in CURD groups decreased this duration ratio (*p* < 0.0001, *n* = 20; Fig. 3j). The ratio of duration time in the empty chamber (the social related three-chamber test) significantly decreased in CURD group while increasing in CUMR and COR groups, with no difference in AMP group; treatments of CURD with Li^+^ or VPA increased the ratio of duration time in empty and stranger chambers (*p* < 0.0001, *n* = 20; Fig. 3k). Again, there was no difference between control and AMP groups (Fig. 3j, k).

Rodents have the propensity to gnaw [31]. We weighed the gnawed scrap of stick during darkness to determine the biting preference of mice (Fig. 3l). The representative images of stick scrap gnawed by mice from different groups in 120 min are shown in Fig. 3l. As compared with control group, the weight of scrap was significantly increased in CURD and AMP groups, but was reduced in CUMR and COR groups (Fig. 3m). The CURD group treated with Li^+^ or VPA significantly decreased this chip weight (Fig. 3m). In the pentobarbital-induced sleep test, the time of sleep latency in six experimental groups were significantly higher than in control group (Fig. 3n), whereas the sleep duration time in these six experimental groups was lower than in control (Fig. 3o). Similarly, the total sleep time without any interference was shortened in all experimental groups (*p* < 0.0001, *n* = 20; Fig. 3p). When compared with CURD group, the administration of Li^+^ and VPA in CURD groups decreased the time of sleep latency (Fig. 3n) and increased the sleep duration and total time (Fig. 3o, p).

### Glymphatic system

**Fig. 4:**
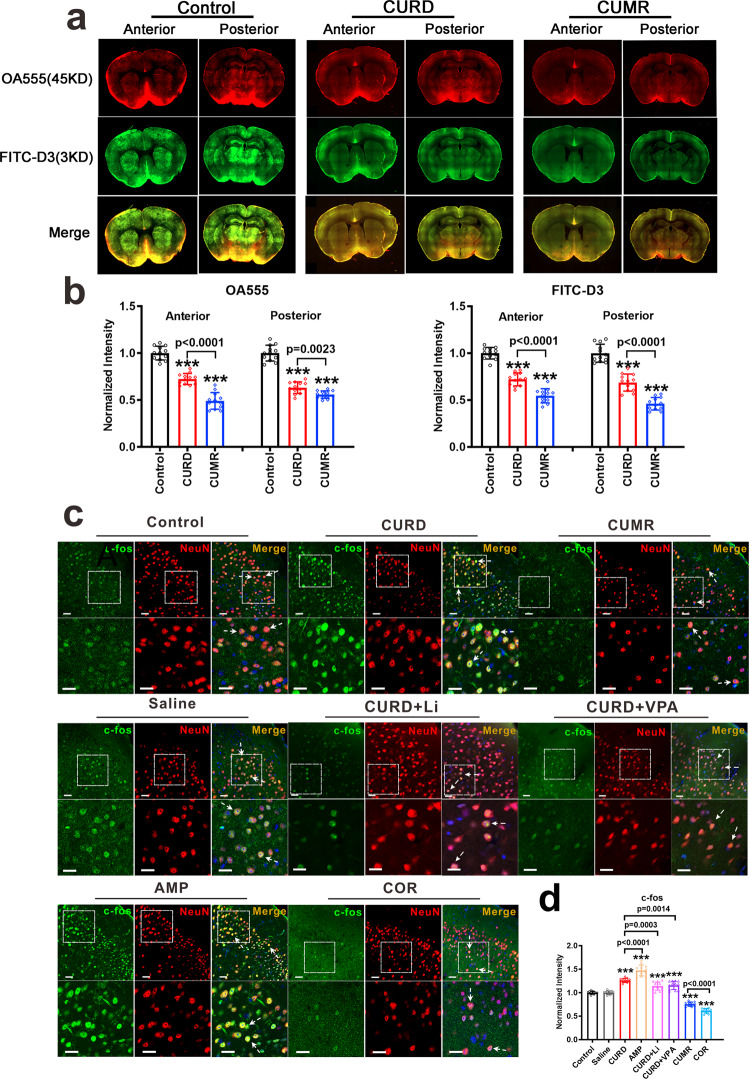
Functional characterisation of the glymphatic system and the neuronal signalling indicators in CURD and CUMR mice models. **a**, **b** A large molecular weight tracer (OA555, 45 kDa) and small molecular weight tracer (FITC-D3, 3 kDa) were injected into cisterna magna. Thirty minutes after injection, the animals were perfusion fixed and the whole-slice fluorescence was measured. **a** The representative images indicate the CSF tracer penetration into the brain, OA555 (red) and FITC-D3 (green). **b** Fluorescence intensities of OA555 and FITC-D3 normalised to the intensity of the control group. **c** The representative images of fluorescence in the frontal cortex, *c-fos* (green), NeuN (red), and DAPI (blue). **d** The intensities of *c-fos* normalised to the intensity of the control group. Data were presented as mean ± SD, *n* = 12 per group. One-way ANOVA for comparisons including more than two groups; unpaired two-tailed *t*-test for two group comparisons. Significant different from control group: ***p < 0.001.

Previously we demonstrated impairment of the glymphatic system in the CUMS mouse depression model [12, 14]. We, therefore, assessed the glymphatic system in CURD and CUMR mice, as shown in Figs. 4a and 4b. We monitored the distribution of fluorescence tracers OA555 (45 kDa) and FITC-D3 (3 kDa) after injecting them into the posterior cistern. Fluorescence intensities of OA555 and FITC-D3 were both significantly reduced in CURD and CUMR-treated mice as compared with the control group (*p* < 0.0001, *n* = 12). At the same time, the intensities of tracers in the CURD group were significantly higher than in the CUMR group (*p* < 0.0001, *p* = 0.0023, *p* < 0.0001, *p* < 0.0001, *n* = 12).

### Neuronal signalling indicators

Protein expression of *c-fos* in cortical neurones may indirectly report neuronal activity [32]. Expression of *c-fos* in neurones was increased in CURD and AMP groups (*p* < 0.0001, *n* = 12; Fig. 4c, d), while decreasing in CUMR and COR groups (*p* < 0.0001, *n* = 12; Fig. 4d), administration of Li^+^ and VPA in CURD groups effectively decreased the levels of *c-fos* (Fig. 4d). Extracellular 5-HT measured by microdialysis increased in CURD and AMP groups (*p* < 0.0001, *n* = 12) and decreased in CUMR and COR groups (*p* < 0.0001, *n* = 12, Supplementary Fig. 2a). Elevated 5-HT in CURD-subjected animals was suppressed by Li^+^ or VPA (Supplementary Fig. 2a). Serotonin transporter SERT/SLC6a4 responsible for 5-HT clearance is linked to depressive or manic symptoms [33–35]. Protein expression of SERT decreased in CURD and AMP groups (*p* < 0.0001, *n* = 12; Supplementary Fig. 2b,c), while it increased in CUMR and COR groups (Supplementary Fig. 2c). Treatment of CURD-exposed mice with Li^+^ and VPA significantly elevated expression of SERT (*p* < 0.0001, *n* = 12, Supplementary Fig. 2c).

### Molecular indicators

**Fig. 5:**
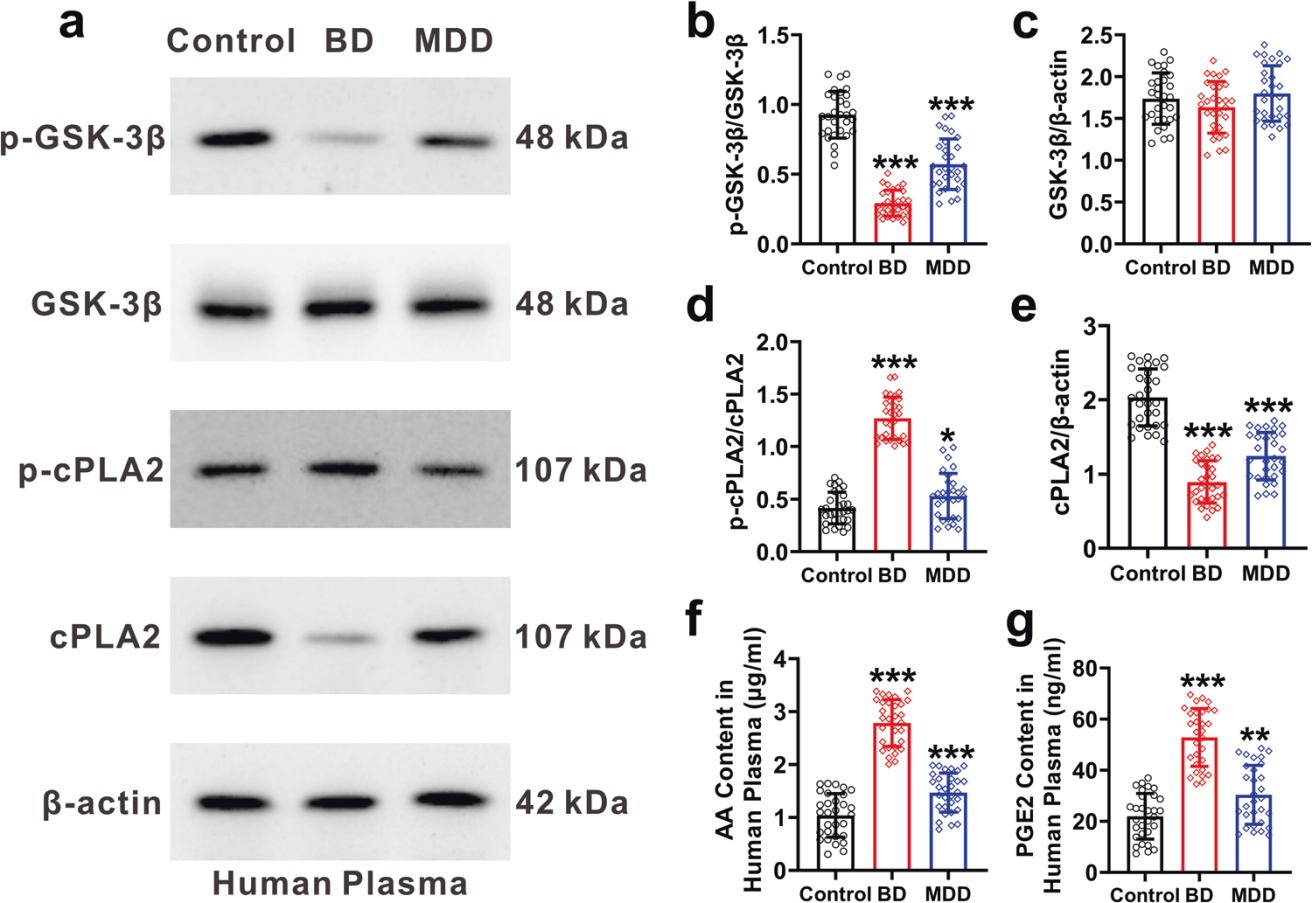
Clinical studies of healthy subjects and patients diagnosed with BD and MDD. **a**–**e** The representative protein bands of p-GSK3β, GSK3β, p-cPLA2, cPLA2, and β-actin from the plasma of healthy subjects and the patients of BD and MDD, and their grey values of protein brands normalised to β-actin. **f** Levels of AA in the plasma of healthy subjects and BD and MDD patients. **g** Levels of PGE2 in the plasma of healthy subjects and patients of BD and MDD. Data were presented as mean ± SD, *n* = 30 per group. One-way ANOVA for comparisons including more than two groups; unpaired two-tailed *t*-test for two group comparisons. Significant different from control group: *p < 0.05, **p < 0.01 and ***p < 0.001.

We collected plasma from the healthy subjects and patients diagnosed with BD and MDD to measure the levels of the phosphorylated (p-GSK3β) and non-phosphorylated GSK3β at ser 9 (inhibitory site), as well as phosphorylated (at ser 505) and non-phosphorylated Ca^2+^-dependent phospholipase A2 (p-cPLA2 and cPLA2 respectively), AA and PGE2. The ratio of p-GSK3β and GSK3β decreased in the plasma of BD and MDD patients when compared with healthy subjects (*p* < 0.0001, *n* = 30, Fig. 5a, b). Levels of GSK3β in the plasma of healthy subjects showed no significant difference from the patients of BD and MDD (*p* = 0.1311, *n* = 30, Fig. 5c). As compared with healthy subjects, the ratio of p-cPLA2 and cPLA2 increased in the plasma of BD and MDD patients (Fig. 5d), whereas the expression of cPLA2 decreased in patients plasma (*p* < 0.0001, *n* = 30, Fig. 5e). The levels of AA and PGE2 were increased in the plasma of BD and MDD patients when compared with healthy subjects (Fig. 5f, g).

**Fig. 6:**
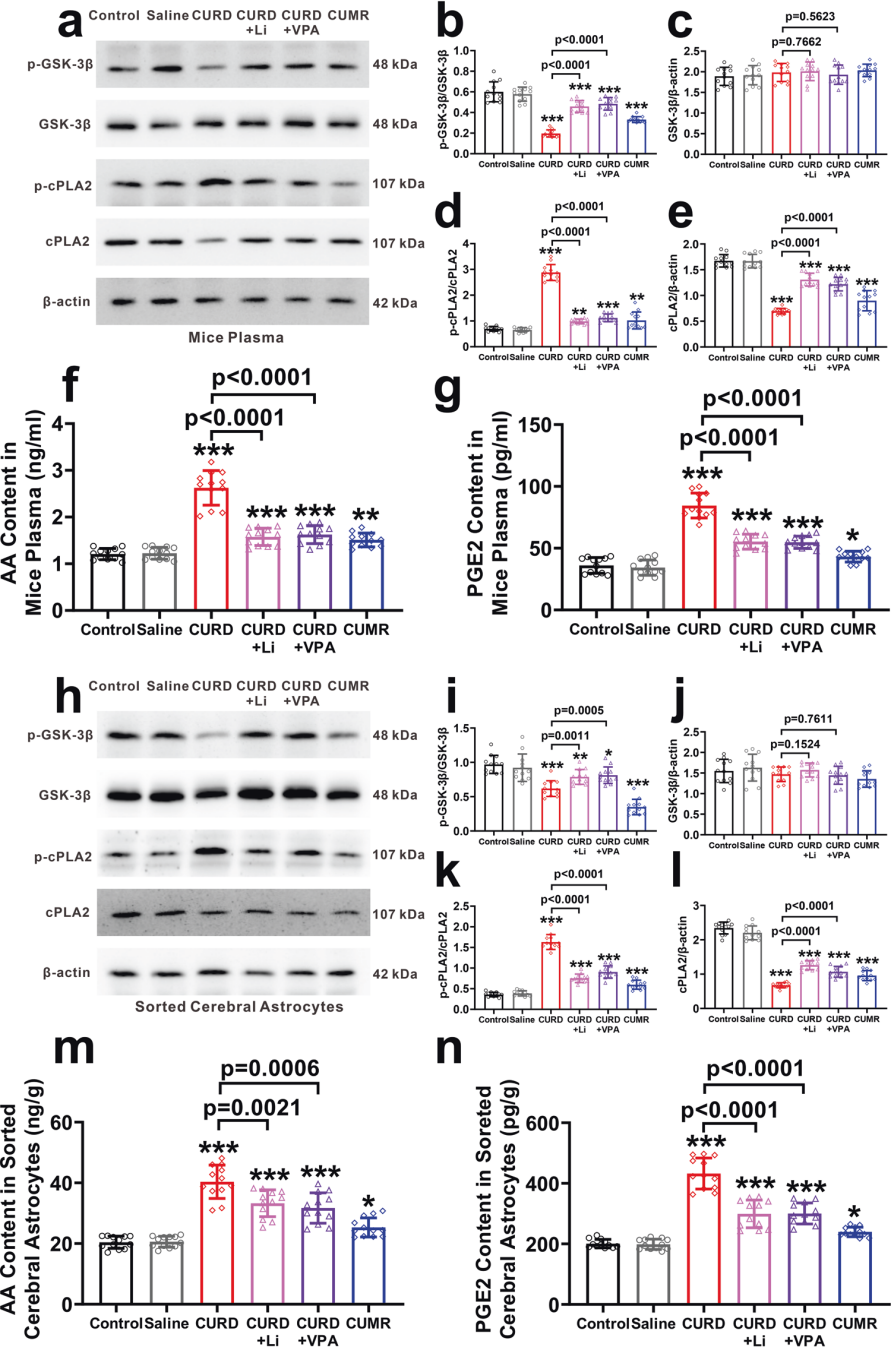
Molecular indicators in the plasma of model mice and cerebral astrocytes. **a**–**e** Representative protein bands of p-GSK3β, GSK3β, p-cPLA2, cPLA2, and β-actin from the plasma of model mice, and the grey values of protein bands normalised to β-actin. **f**, **g** The levels of AA and PGE2 in the plasma of model mice. **g** The levels of AA in the plasma of model mice. **h**–**l** Representative protein bands of p-GSK3β, GSK3β, p-cPLA2, cPLA2, and β-actin from the sorted astrocytes of Aldh1l1-EGFP, and the grey values of protein bands normalised to β-actin. **m**, **n** Levels of AA and PGE2 in cortical astrocytes. Data were presented as mean ± SD, *n* = 12 per group. One-way ANOVA for comparisons including more than two groups; unpaired two-tailed *t*-test for two group comparisons. Significant different from control group: *p < 0.05, **p < 0.01 and ***p < 0.001.

Subsequently, we measured these indicators in the plasma and in the FAX-sorted cerebral astrocytes of manic model mice (Fig. 6). When compared with the control group, the ratio of p-GSK3β and GSK3β was significantly decreased in both the plasma and cerebral astrocytes of CURD group and CUMR group (Fig. 6a, b, h, i). Expression of GSK3β in plasma and astrocytes showed no difference between groups (Fig. 6c, j). As compared with the control group, the ratio of p-cPLA2 and cPLA2 significantly increased in the cerebral astrocytes and plasma of the CURD and CUMR group (Fig. 6d, k), whereas the expression of cPLA2 was significantly reduced in CURD and in CUMR groups (Fig. 6e, l); Treatments of CURD group with Li^+^ or VPA restored the ratio of p-GSK3β and GSK3β as well as p-cPLA2 and cPLA2 to the control values (Fig. 6b, i, d, k), and increased the expression of cPLA2 (Fig. 6e, l). As compared with the control group, the levels of AA and PGE2 were significantly increased in the plasma and cerebral astrocytes of the CURD and CUMR groups group; the treatment with Li^+^ and VPA effectively suppressed increased AA and PGE2 in cerebral astrocytes and plasma of CURD groups (Fig. 6f, g, m, n).

We further analysed clinical and experimental data with Spearman correlation analysis. For the five molecular indicators, including the ratio of p-GSK3β and GSK3β, the ratio of p-cPLA2 and cPLA2, cPLA2 and the levels of AA and PGE2, we found significant positive correlations between plasma of BD patients and plasma of CURD mice (0.5 < *r* < 1, *p* < 0.05, Supplementary Fig. 3a); similar correlations were also observed between plasma of MDD patients and plasma of CUMR mice (0.5 < *r* < 1, *p* < 0.05, Supplementary Fig. 3a). Likewise, these five indicators demonstrated positive correlations between plasma of CURD mice and cerebral astrocytes of CURD-model mice or between plasma of CUMR mice and cerebral astrocytes of CUMR model mice (0.5 < *r* < 1, *p* < 0.05, Supplementary Fig. 3b).

## Discussion

We developed treatment regimens to establish divergent mice models replicating manic or depressive syndromes. Mice exposed to the CURD regimen present signs of manic behaviour. These animals are hyperactive in the home cages (Fig. 1a) and in the open field (Fig. 1j–l), they exhibit reduced immobility time in tail suspension and forced swimming tests (Fig. 1h, i), and their exploratory behaviours (Fig. 1n), as well as social interactions (Fig. 2a–e), are increased. These signs were accompanied by a disturbed rhythm of sleep (Fig. 2f, g) and shortened sleep duration (Fig. 2h). Similar behavioural manifestations, as well as sleep disturbances and increased social presence, are observed in humans during manic episodes [36–38]. Besides establishing an effective behavioural manic model, we produced an improved depression model using the newly developed CUMR protocol. Mice exposed to CUMR protocol display depressive-like behaviours. These behaviours are, in general, similar to that observed in other manic models and comparable to the clinical presentations of mood disorders with manic or depressive components (Supplementary Table 3).

The CURD-model of mania is more comprehensive than the pharmacological (amphetamine) model. It is well documented that in healthy humans, 20 mg of amphetamine increase motor activity without increasing specific exploration and without marked effects on spatial patterns of activity [39]. The amphetamine-induced behaviours are inconsistent with behavioural presentations observed in the manic phase of bipolar disorder, which is characterised by both hyperactivity and increased exploration [40–43]. Conversely, animals injected with amphetamine to model mania present hyperactivity alone with no effect on exploratory behaviour [44].

Nearly all patients suffering from mood disorders have significant disruptions in circadian rhythms and the sleep/wake cycle [45], with altered sleep patterns being of the major diagnostic value. Environmental disruptions of circadian rhythms, including shift work, travel across time zones, and irregular social schedules, tend to precipitate or exacerbate mood-related symptoms of mania or the manic episodes of BD [46]. Seasonal alternations are similarly linked to the occurrence of mania, with manic episodes being more frequent in spring and summer. In contrast, depressive episodes peak in early winter and are less frequent in summer [47]. Moreover, the distinctive cyclicity between manic and depressive episodes are very difficult to reproduce in mice; neither pharmacological nor genetic animal models of mania include similar associations. In the CURD protocol, normal rhythms are disturbed by eight interventions (Supplementary Fig. 1b–i).

Besides the manic-like behaviours, CURD-treated mice also had functional abnormalities. The glymphatic system is malfunctional in CURD and CUMR groups (Fig. 4a). The *c-fos* as a broad indicator of neuronal activity was increased in CURD and AMP-treated mice (Fig. 4c), while the expression of SERT in the cortex was significantly reduced in CURD and AMP groups, being associated with the elevated extracellular 5-HT (Supplementary Fig. 2b,c). The depression-related CUMR or COR regimens demonstrated the opposite changes in *c-fos*, SERT and 5-HT compared with CURD and AMP groups (Fig. 4c, d and Supplementary Fig. 2a–c). Some of the changes outlined above were reported in mania patients. For example, the level of 5-HT metabolite 5-hydroxyindoleacetic acid (5-HIAA) is elevated in cerebrospinal fluid (CSF) in 14 hospitalised drug-free manic patients [48, 49].

AA is implicated in BD, MDD and the risk of suicide [50–52]. The cPLA2 hydrolyses the acyl bond of AA from the *sn-2* position in the cell membrane, thus allowing AA conversion to prostaglandins such as PGE2 by cyclooxygenase-2 (COX-2) and prostaglandin E synthase (PGES) [53]. As demonstrated previously, cPLA2 is selectively changed in the cerebral astrocytes from the CUMS model mice, while antidepressants specifically regulate the expression of astrocytic cPLA2 [13, 54]. Recently reported GSK3β polymorphisms are associated with the high risk of MDD in the Chinese Han Population [55]. We, therefore, compared these molecular indicators (GSK3β, cPLA2, AA and PGE2) between patients of BD and MDD and mouse manic model.

Although the reported levels of AA in patients' blood vary substantially, recent reports highlight the association between high AA levels and depression severity, whereas a low level of AA may predict subsequent suicide attempts [50]. We confirmed increased levels of AA in the plasma of MDD or BD patients. Treatment with Li^+^ and VPA effectively restored molecular indicators and improved the behavioural performances of CURD-treated mice. We demonstrated previously, that chronic treatment with Li^+^ or VPA significantly decreased the mRNA and protein expression of cPLA2 in the primary cultured astrocytes [56]. Additionally, activation of GSK3β was increased in CURD and CUMR model mice, with similar trends in BD and MDD patients' plasma. Similarly, increased GSK3β activity in the platelets was suggested to associate with the pathophysiology of both depressive disorder and neurodegenerative diseases [57].

## Conclusions

Mania and the manic episodes of BD are serious mental disorders. The shortage of appropriate animal models severely hinders the advances in research on pathogenesis and pharmacological management of mania. We designed the novel mania model induced solely by environmental disturbances, which are easy to replicate. The biological indicators measured in this novel manic model mice agree well with the clinical data obtained from patients with mood disorders. Specific treatment with anti-manic drugs, effectively modified the disease-related indicators and behavioural performance. This novel model of mania will aid research into fundamental and applied aspects of the manic syndrome.

### Supplementary information

Supplementary information summary

Supplementary Table 1

Supplementary Table 2

Supplementary Table 3

Supplementary Figure 1

Supplementary Figure 2

Supplementary Figure 3

Supplementary figure legends

Supplementary Video 1

Supplementary Video 2

Supplementary Video 3

Supplementary Data 1

Supplementary Data 2

Supplementary Data 3

## Data Availability

Source data and statistical analysis underlying the main and supplementary figures are available in Supplementary Data 2.
