# Supplementary material for: A novel murine model of mania
Source: Mol Psychiatry. 2023 Mar 29;28(7):3044–54. doi: 10.1038/s41380-023-02037-8 (PMC10615760; doi:10.1038/s41380-023-02037-8)
Supplement: Supplementary file 1 — Supplementary information summary [file 41380_2023_2037_MOESM1_ESM.doc]

**Supplementary Information Summary**

**Supplementary Table 1: The details of treatments with CURD and CUMR in three weeks.**

**Supplementary** **Table 2: The demographics of health subjects and patients.**

**Supplementary Table 3: The potential correspondence between the behavioral performance of model mice and the clinical symptoms of patients.**

**Supplementary Figure 1: The protocol and stressors used for chronic unpredictable rhythm disturbance (CURD) and chronic unpredictable mild restraint (CUMR).**

**Supplementary Figure 2: The levels of extracellular 5-HT and SERT in mice model of CURD or CUMR treated with specific drugs.**

**Supplementary Figure 3: Correlation analysis of functional and molecular indicators.**

**Supplementary figure legends.**

**Supplementary Video 1: The representative performance of a mouse in control group.**

**Supplementary Video 2: The representative performance of a mouse in CURD group.**

**Supplementary Video 3: The representative performance of a mouse in CUMR group.**

**Supplementary Data 1: the details of methods and materials.**

**Supplementary Data 2: the original data and statistical analysis of the main and supplementary figures.**

**Supplementary Data 3: the original whole gels of western blotting bands.**
