# Supplementary material for: A novel murine model of mania
Source: Mol Psychiatry. 2023 Mar 29;28(7):3044–54. doi: 10.1038/s41380-023-02037-8 (PMC10615760; doi:10.1038/s41380-023-02037-8)
Supplement: Supplementary file 2 — Supplementary Table 1 [file 41380_2023_2037_MOESM2_ESM.doc]

**Supplementary Table 1. The details of treatments with CURD and CUMR in three weeks.**

| Date | | CURD Treatment | | CUMR Treatment | |
| --- | --- | --- | --- | --- | --- |
| 1 Week | 1d | CRI (Model 1) | Noise disturbance | Restraint | Damp sawdust |
|  | 2d | SD | High temper stress | Cage shaking | Tail suspension |
|  | 3d | OSCLTI | Foot shock | Forced swimming | 45° Cage tilting |
|  | 4d | OSAF | Noise disturbance | Restraint | Tail suspension |
|  | 5d | High temper stress | Stroboscopic illumination | Damp sawdust | Cage shaking |
|  | 6d | CRI (Model 2) | SD | Forced swimming | Tail suspension |
|  | 7d | OSCLTI | Noise disturbance | 45° Cage tilting | Cage shaking |
| 2 Week | 8d | OSAF | High temper stress | Restraint | Tail suspension |
|  | 9d | SD | Foot shock | 45° Cage tilting | Damp sawdust |
|  | 10d | Stroboscopic illumination | Noise disturbance | Forced swimming | Cage shaking |
|  | 11d | CRI (Model 1) | High temper stress | Restraint | Tail suspension |
|  | 12d | SD | Foot shock | 45° Cage tilting | Damp sawdust |
|  | 13d | OSAF | CRI (Model 2) | Forced swimming | Cage shaking |
|  | 14d | High temper stress | Noise disturbance | Damp sawdust | Cage shaking |
| 3 Week | 15d | Stroboscopic illumination | Foot shock | Restraint | 45° Cage tilting |
|  | 16d | CRI (Model 2) | Noise disturbance | Forced swimming | Tail suspension |
|  | 17d | OSCLTI | SD | Damp sawdust | Cage shaking |
|  | 18d | OSAF | High temper stress | Restraint | Tail suspension |
|  | 19d | Stroboscopic illumination | Noise disturbance | Forced swimming | Tail suspension |
|  | 20d | SD | Foot shock | Damp sawdust | Cage shaking |
|  | 21d | CRI (Model 1) | High temper stress | Restraint | 45° Cage tilting |

CRI: Circadian rhythm interference; SD: Sleep deprivation; OSCLTI: One solid cone light was turned on irregularly; OSAF: One spotlight always followed the mice for 12 hours.
