# Supplementary material for: A novel murine model of mania
Source: Mol Psychiatry. 2023 Mar 29;28(7):3044–54. doi: 10.1038/s41380-023-02037-8 (PMC10615760; doi:10.1038/s41380-023-02037-8)
Supplement: Supplementary file 3 — Supplementary Table 2 [file 41380_2023_2037_MOESM3_ESM.doc]

**Supplementary** **Table 2. The demographics of health subjects and patients.**

| Variable | HS | BD | MDD |
| --- | --- | --- | --- |
| Age(years), mean ± SD | 37.03±8.17 | 32.90±9.36 | 34.70±8.05 |
| Gender | 13M, 17F | 18M, 12F | 11M, 19F |
| Race | 30 Han | 30 Han | 30 Han |
| HDRS | - | 16.33±9.53 | 28.77±9.81 |
| CARS-M | - | 15.40±9.52 | 4.07±2.05 |
