# Supplementary material for: A novel murine model of mania
Source: Mol Psychiatry. 2023 Mar 29;28(7):3044–54. doi: 10.1038/s41380-023-02037-8 (PMC10615760; doi:10.1038/s41380-023-02037-8)
Supplement: Supplementary file 4 — Supplementary Table 3 [file 41380_2023_2037_MOESM4_ESM.doc]

**Supplementary** **Table 3: The potential correspondence between the behavioral performance of model mice and the clinical symptoms of patients.**

|  | CURD | AMP | CUMR | COR | BD | MDD |
| --- | --- | --- | --- | --- | --- | --- |
| Sucrose Preference Test - Sucrose Preference (%) | ↓ | ↓ | ↓ | ↓ | pleasure loss | pleasure loss |
| Sucrose Preference Test - Total Consumption (ml) | ↑ | ↑ | ↓ | ↓ | appetite increase | appetite decrease |
| Sucrose Pellets Preference Test - Sucrose Pellets Preference (%) | ↓ | ↓ | ↓ | ↓ | pleasure loss | pleasure loss |
| Sucrose Pellets Preference Test - Total Consumption (g) | ↑ | ↑ | ↓ | ↓ | appetite increase | appetite decrease |
| Tail Suspension Test - Immobility Time (s) | ↓ | ↓ | ↑ | ↑ | energy increase | hopeless; energy loss |
| Forced Swimming Test - Immobility Time (s) | ↓ | ↓ | ↑ | ↑ | energy increase | hopeless; energy loss |
| Open Field Test -Total Distance (m) | ↑ | ↑ | ↓ | ↓ | psychomotor agitation | psychomotor retardation |
| Open Field Test - Time Spent in Central (s) | ↑ | — | ↓ | ↓ | psychomotor agitation | psychomotor retardation |
| Open Field Test - Vertical Counts (time) | ↑ | — | ↓ | ↓ | psychomotor agitation | psychomotor retardation |
| Three-chamber Sociability Test - Social Circle Duration Rate (%) | ↑ | — | ↓ | ↓ | goal-directed activity increase | social barriers |
| Three-chamber Sociability Test - Chamber Duration Rate (%) - Empty | ↓ | — | ↑ | ↑ | goal-directed activity increase | social barriers |
| Three-chamber Sociability Test - Chamber Duration Rate (%) - Stranger | ↑ | — | ↓ | ↓ | goal-directed activity increase | social barriers |
| Bite Ability Test - Chippings Weight (g) | ↑ | ↑ | ↓ | ↓ | psychomotor agitation | psychomotor retardation |
| Pentobarbital-induced Sleep Test - Sleep Latency (s) | ↑ | ↑ | ↑ | ↑ | sleep disturbance | sleep disturbance |
| Pentobarbital-induced Sleep Test - Sleep Duration (min) | ↓ | ↓ | ↓ | ↓ | sleep disturbance | sleep disturbance |
| Observed Sleep Test - Total Sleep Time (h) | ↓ | ↓ | ↓ | ↓ | sleep disturbance | sleep disturbance |
