# Supplementary figures and images for: A novel murine model of mania
Source: Mol Psychiatry. 2023 Mar 29;28(7):3044–54. doi: 10.1038/s41380-023-02037-8 (PMC10615760; doi:10.1038/s41380-023-02037-8)

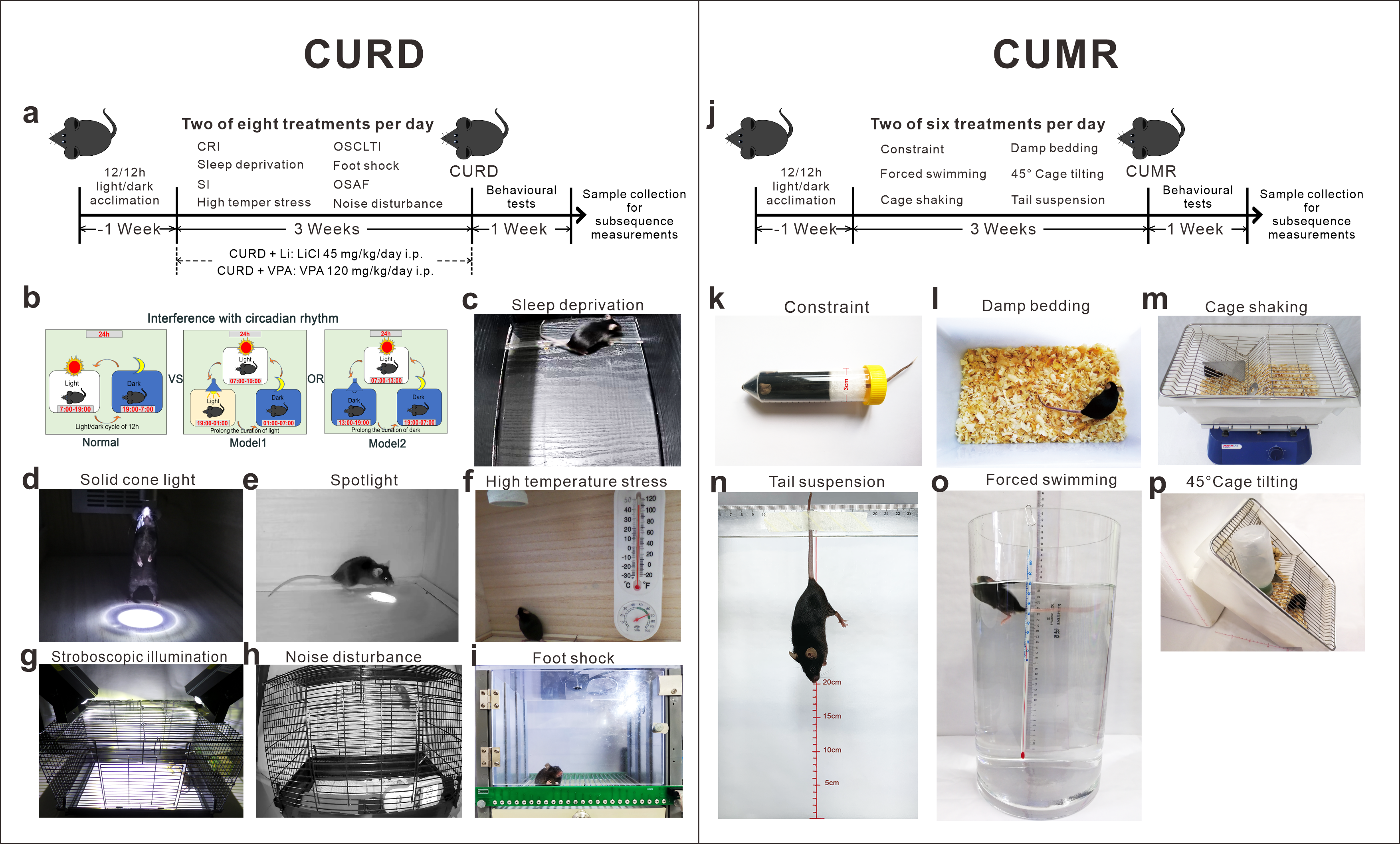

Supplement: Supplementary file 5 — Supplementary Figure 1 [file 41380_2023_2037_MOESM5_ESM.tif]

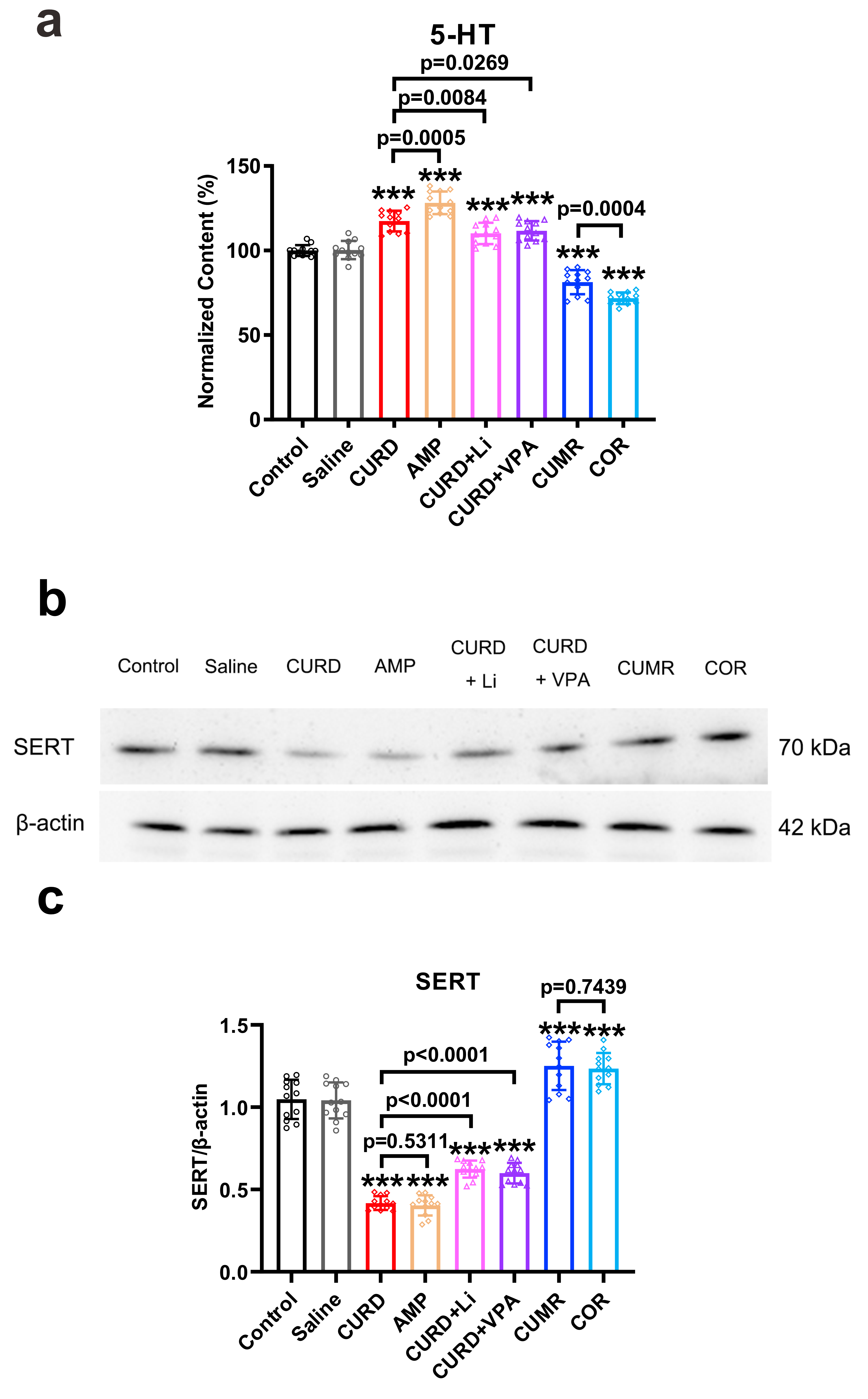

Supplement: Supplementary file 6 — Supplementary Figure 2 [file 41380_2023_2037_MOESM6_ESM.tif]

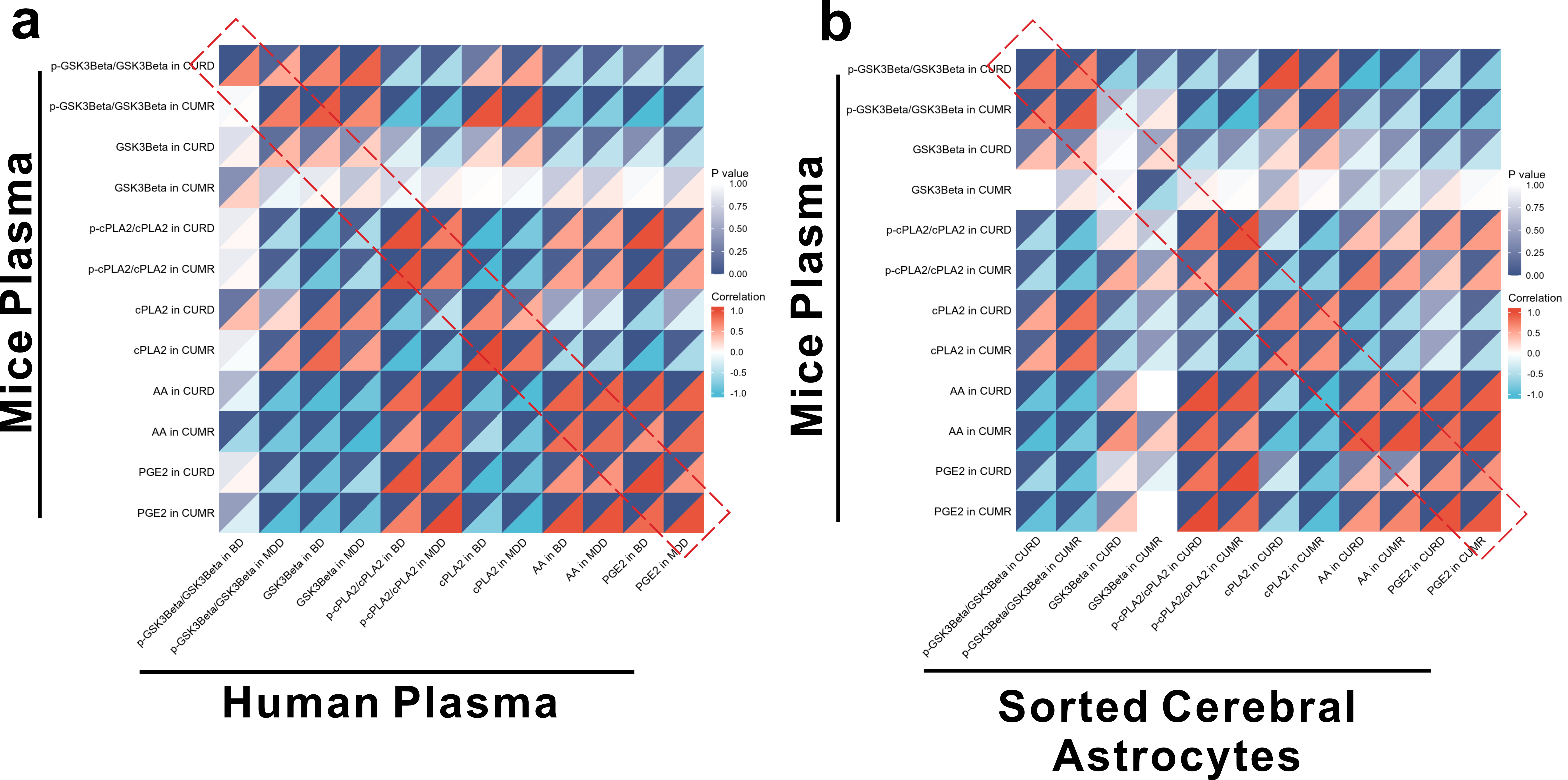

Supplement: Supplementary file 7 — Supplementary Figure 3 [file 41380_2023_2037_MOESM7_ESM.tif]

**Supplementary Data 3: the original whole gels of western blotting bands**

**
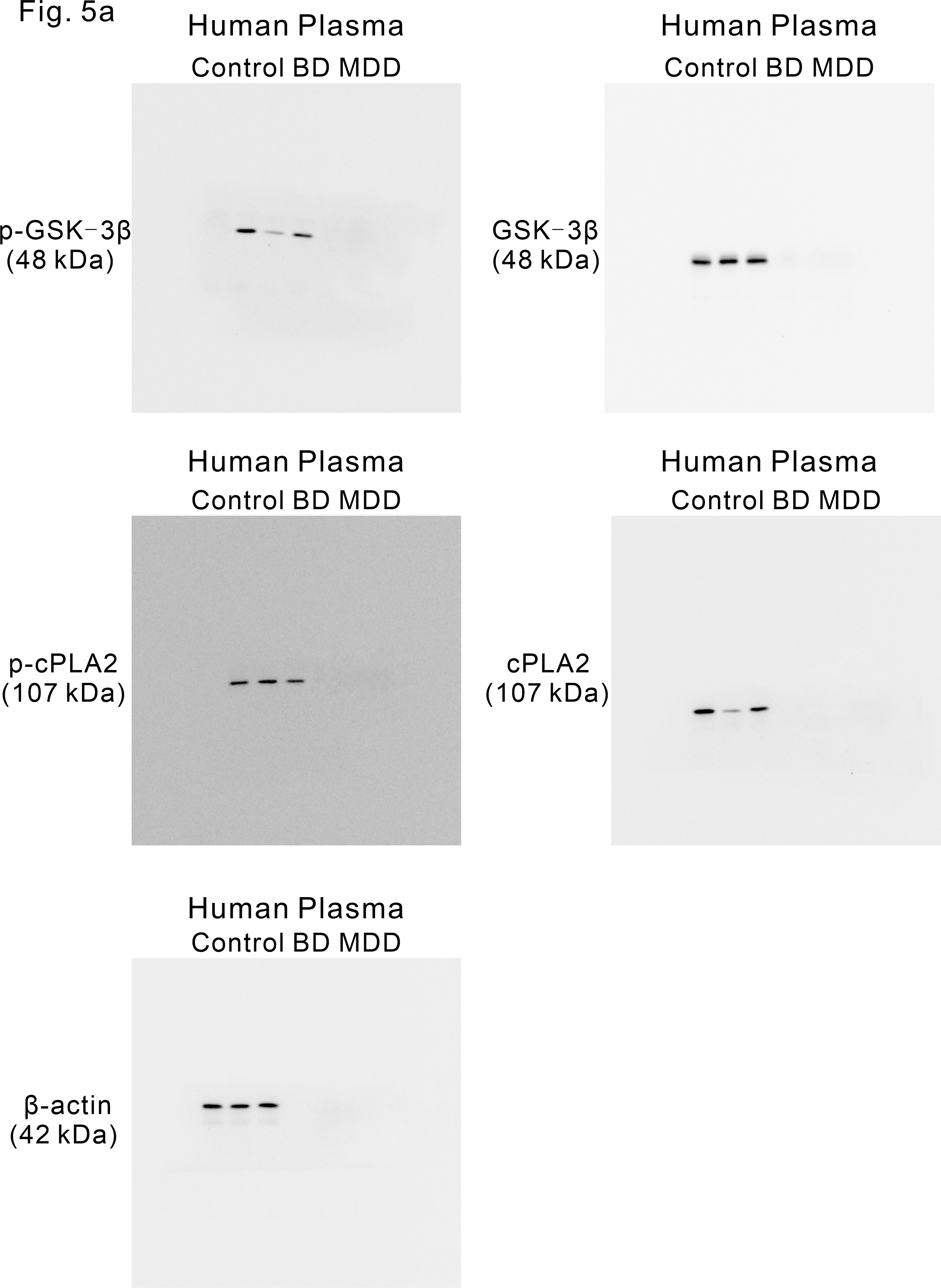

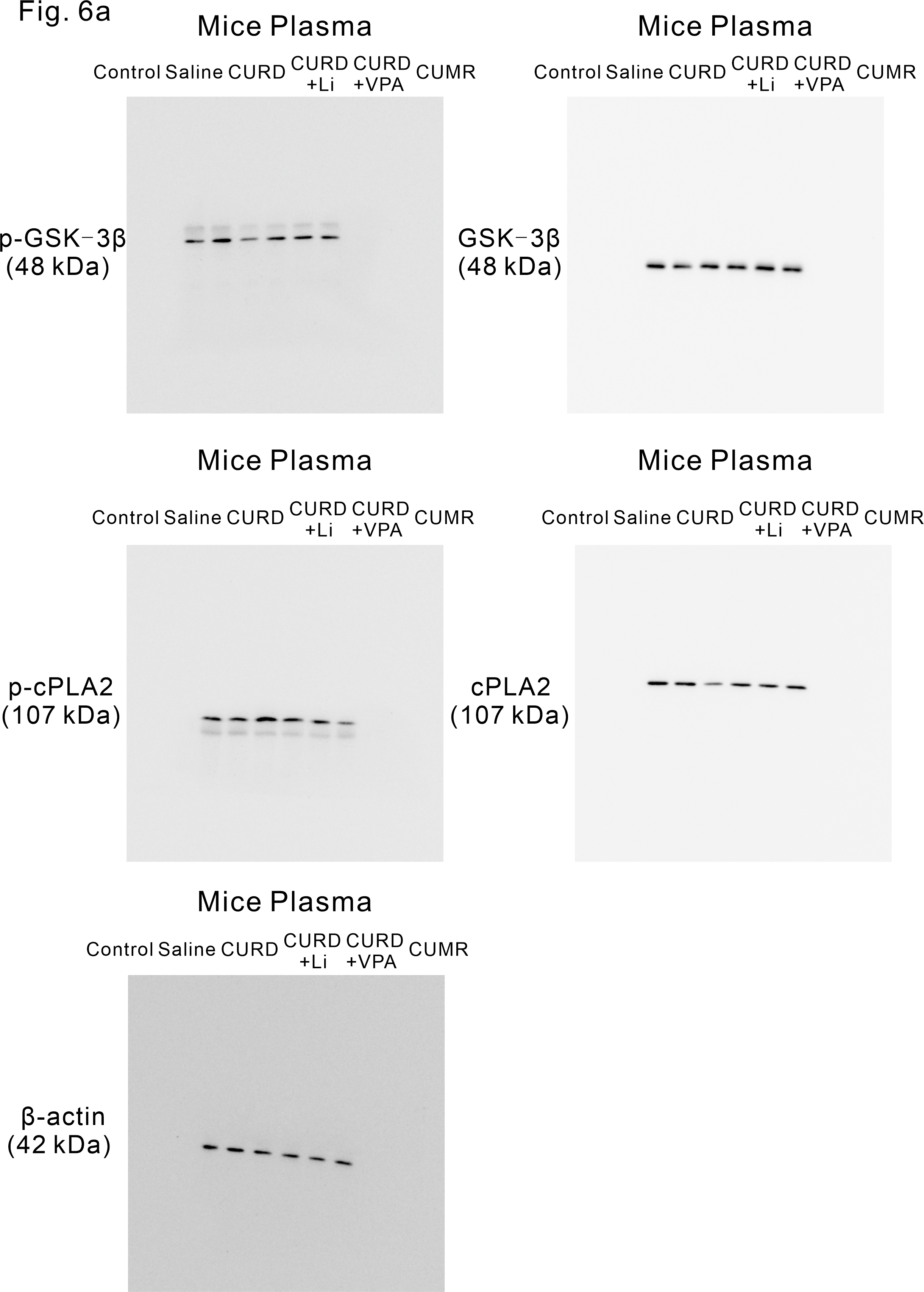

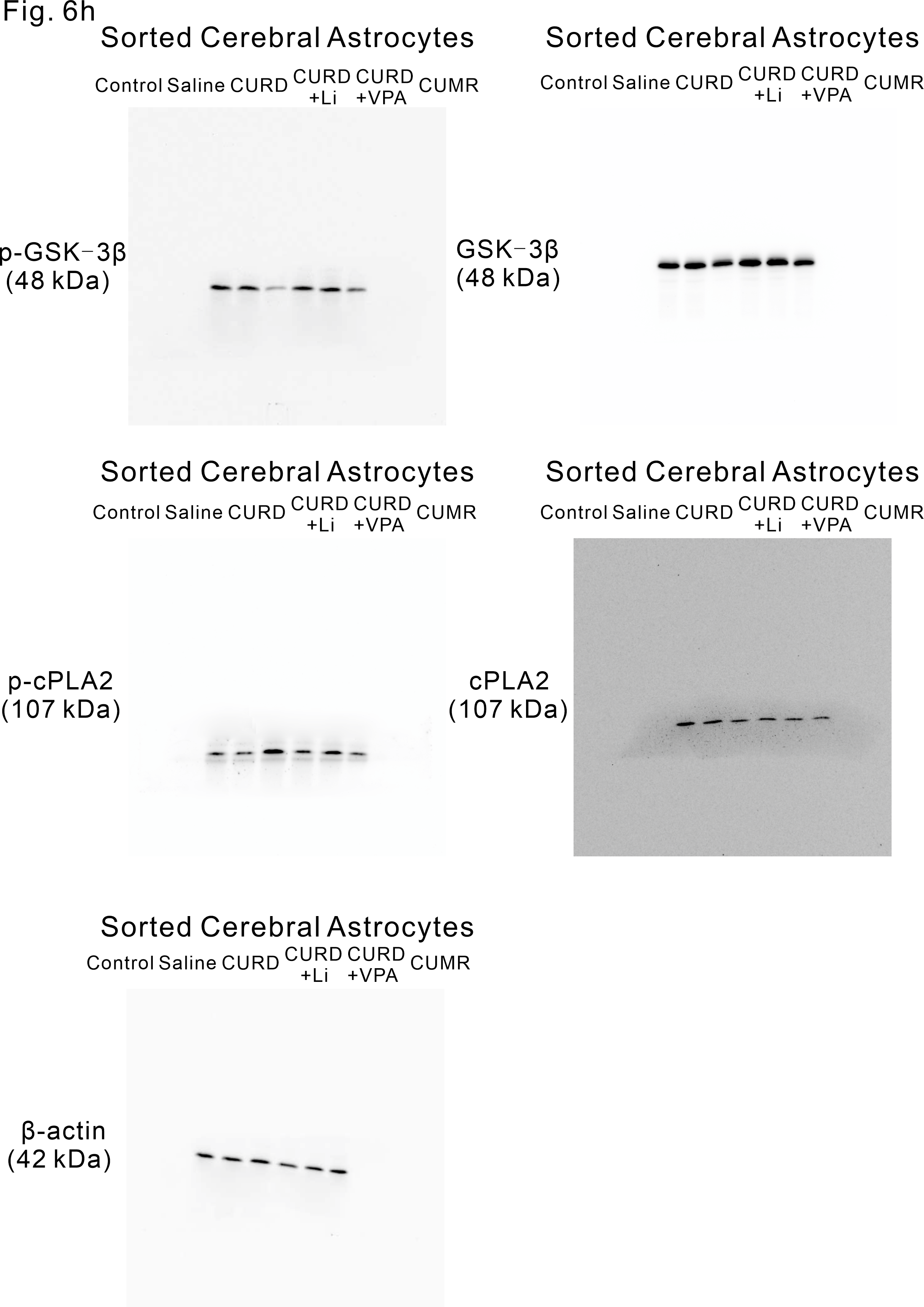

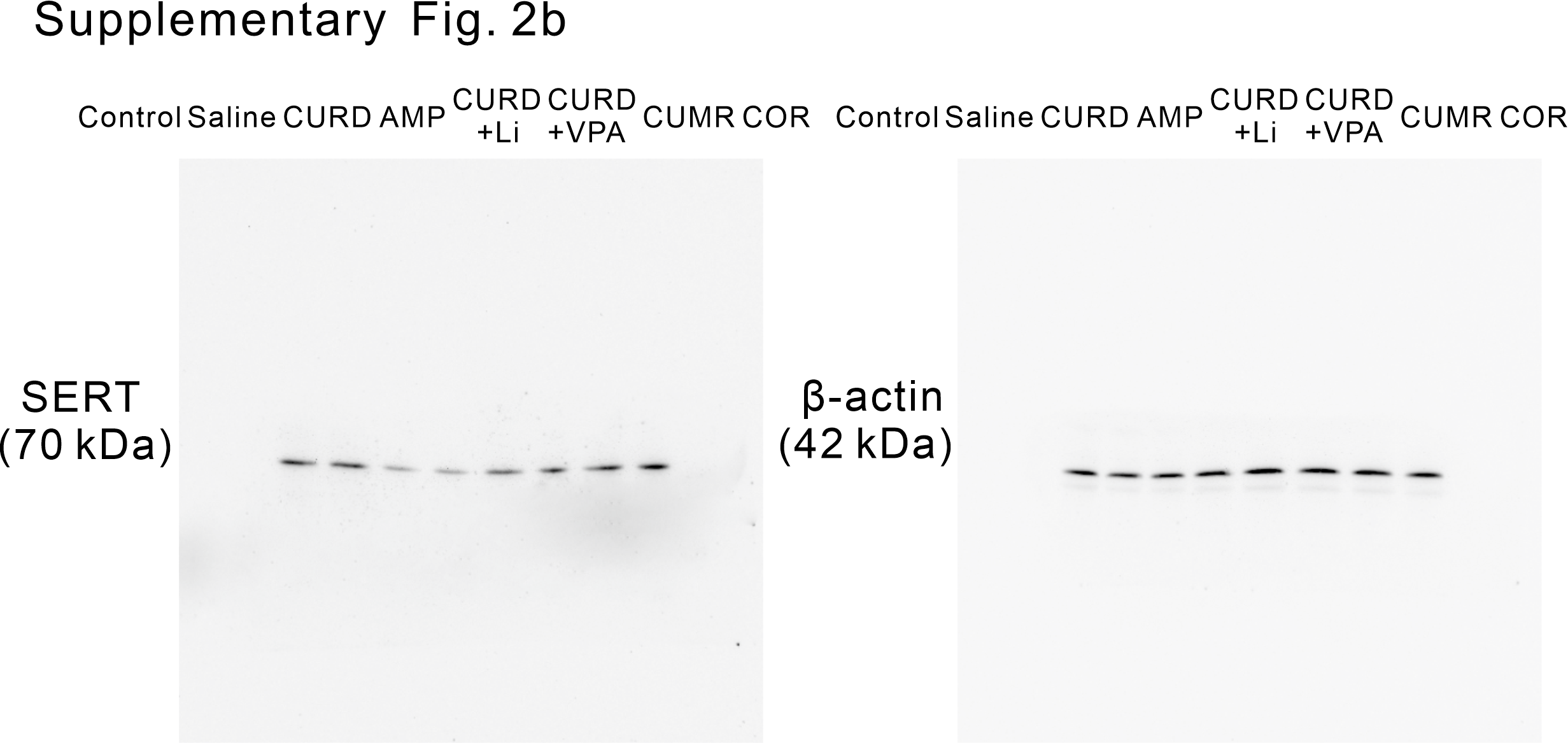
**

Supplement: Supplementary file 14 — Supplementary Data 3 [file 41380_2023_2037_MOESM14_ESM.doc]
