# Supplementary material for: A novel murine model of mania
Source: Mol Psychiatry. 2023 Mar 29;28(7):3044–54. doi: 10.1038/s41380-023-02037-8 (PMC10615760; doi:10.1038/s41380-023-02037-8)
Supplement: Supplementary file 8 — Supplementary figure legends [file 41380_2023_2037_MOESM8_ESM.doc]

**Supplementary Figure Legends**

**Supplementary Figure 1. The protocol and stressors used for chronic unpredictable rhythm disturbance (CURD) and chronic unpredictable mild restraint (CUMR).**

To establish CURD model, the interference of circadian rhythm (b), sleep deprivation (c), the interference of cone light (d), the interference of followed spotlight (e), high temperature stress (f), stroboscopic illumination (g), noise disturbance (h) and foot shock (i) were employed. Two out of these eight stressors were randomly chosen and applied every day, the whole period of disturbed rhythm was 3 weeks. The behavioural tests were operated in the last one week, then the samples were collected and used in the measurements. To establish CUMR model, constraint (k), damp bedding (l), cage shaking (m), tail suspension (n), forced swimming (o) and cage tilting (p) were used. Two out of these six behavioral constraints were randomly selected and applied every day for 3 weeks.

**Supplementary Figure 2. The levels of extracellular 5-HT and SERT in mice model of CURD or CUMR treated with specific drugs.**

(a) The extracellular 5-HT level normalised by control group in frontal cortex. (b) The representative protein bands of SERT and β-actin, and the normalised gray value of SERT protein bands to β-actin were shown in (c). Data are presented as mean ± SD, n=12 per group. One-way ANOVA for comparisons including more than two groups; unpaired two-tailed t-test for two group comparisons. Significant different from control group: *p < 0.05, **p < 0.01, ***p < 0.001.

**Supplementary Figure 3. Correlation analysis of functional and molecular indicators.**

Six indicators, including the ratio of p-GSK3β and GSK-3β, the ratio of GSK-3β and β-actin (GSK-3β), the ratio of p-cPLA2 and cPLA2, the ratio of cPLA2 and β-actin (cPLA2), the levels of AA and PGE2, were used for correlation analysis between the plasma of wild type mice and the plasma of patients and healthy subjects (a) and between the plasma of wild type mice and the sorted astrocytes of Aldh1l1-EGFP mice (b). Each color block is divided into two triangles, the upper triangle represents the p value of statistical analysis. In the lower triangle, red represents positive correlation, blue represents negative correlation, the depth of the color indicates the correlation coefficient, as shown in the gradient color scales.
