# Supplementary material for: A novel murine model of mania
Source: Mol Psychiatry. 2023 Mar 29;28(7):3044–54. doi: 10.1038/s41380-023-02037-8 (PMC10615760; doi:10.1038/s41380-023-02037-8)
Supplement: Supplementary file 12 — Supplementary Data 1 [file 41380_2023_2037_MOESM12_ESM.doc]

**Supplementary Data 1: the details of methods and materials**

**Materials**

Most chemicals, including Alexa 555-conjugated ovalbumin (45 kDa) and Fluorescein 3000 MW Anionic dextran (3 kDa) were purchased from Invitrogen (CA, USA). D-Amphetamine (14204) was purchased from Cayman (Michigan, USA). Pentobarbital sodium (P3761), primary antibody of SERT (SAB2502028), β-actin (A5441), serotonin (14927), lithium chloride (Li^+^; L9650), sodium valproate (VPA; P4543) and corticosterone (27840) were purchased from Sigma (MO, USA). Primary antibody of NeuN (PA5-78693) was purchased from Thermo Fisher Scientific (Waltham, MA USA). 4,6-diamidine-2-phenylindole dihydrochloride (DAPI), primary antibody of, p-cPLA2 at ser 505 (PA5-17790) and p-GSK3β at ser 9 (MA5-14873) were purchased from Thermo Fisher Scientific (CA, USA). Primary antibody for c-fos (sc-271243) and cPLA2 (sc-454) were purchased from Santa Cruz Biotechnology (Dallas, Texas, USA). ELISA kits of arachidonic acid (AA; E-EL-0051c) and prostaglandin E2 (PGE2; E-EL-0034c) were purchased from Elabscience Biotechnology Co. Ltd (Hubei, China). GSK3β (22104-1-AP) was from Proteintech Group, Inc (IL, USA).

**Animals**

Male wild type C57BL/6 mice (#000664; aged 10 - 12 weeks; weight of 25 - 35 g) and B6-Tg-Aldh1l1-EGFP mice (#030247; aged 10 - 12 weeks; weight of 25 - 35 g) were purchased from the Jackson Laboratory (Bar Harbor, ME, USA). Animals were raised in standard housing conditions (22 ± 1℃; light/dark cycle of 12/12h), with water and food available *ad libitum*. All experiments were performed in accordance with the US National Institutes of Health Guide for the Care and Use of Laboratory Animals (NIH Publication No. 8023) and its 1978 revision, and all experimental protocols were approved by the Institutional Animal Care and Use Committee of China Medical University, No. [2020]102.

**Chronic unpredictable rhythm disturbance (CURD) regimen**

Male mice were exposed to the pattern of stressors for 3 weeks. The normal rhythm was alternated 12 hours between daily light (7:00-19:00) and darkness (19:00-7:00). The circadian rhythm was interfered with by shortening 6 hours in the darkness time (mode 1) or in the daily light time (mode 2), sleep deprivation (6 hours), stroboscopic illumination (3 watt, 120/min for 12 hours) in the dark, one solid cone light (1 watt) was turned on irregularly during the dark period (12 hours), one spotlight (0.5 watt) always followed the mice during the darkness (12 hours), environmental temperature increase to 40 - 45°C (30 minutes), noise (100 dB for 12 hours), foot shock (0.8 mA for 2 seconds). The recovery time between stressors was at least 6 hours. Experimental mice were individually housed, two of these eight stressors were randomly selected by a random number table and applied once every day, the detailed everyday treatment protocol is summarized in Supplementary Table 1.

**Chronic unpredictable mild restraint (CUMR) regimen**

Male mice were exposed to the following stressors for 3 weeks: restricting activity (4 hours), damp bedding (12 hours), cage shaking (40/minute for 10 minutes), tail suspension (10 minutes), forced swimming (10 minutes 25 °C), 45° tilting cage (12 hours). Two of these six treatments were randomly scheduled once every day, and continued for 3 weeks; detailed everyday treatment protocol is shown in Supplementary Table 1.

**Pharmacological models**

Amphetamine (AMP) was dissolved in normal saline (NS) and intraperitoneally injected at 2.5 mg/kg/day in a volume of 5 ml/kg for 10 days [1]. The corticosterone was dissolved in NS containing 0.1% dimethylsulfoxide (DMSO) and 0.1% Tween-80 and intraperitoneally injected at 20 mg/kg/day in a volume of 5 ml/kg for 28 days [2, 3].

**Ethogram**

The mice status was evaluated according to general appearance parameters (GAP) assessments. A score of 0 or 1 for the categories of activity, posture, breathing pattern, coat condition, and interaction with other mice were given. If the parameter was normal, it was recorded as 0, if the parameter was abnormal, it was recorded as 1. The higher the score, the worse is the state of the mice [4, 5].

**Sucrose preference test**

The sucrose preference is a reward-based test and a measure of anhedonia. At first, test mice were adapted to 2.5% sucrose solution for 48 hours. After 12 hours of food and water deprivation, mice were provided with two pre-weighed bottles, including one bottle that contained 2.5% sucrose solution and a second bottle filled with water, for 12 hours. The total intake volume of pure and sucrose water was calculated, separately. The percentage preference was calculated according to the following formula: % preference = [sucrose intake/ (sucrose + water intake)] × 100% [6-8].

**Sucrose pellets preference test**

The sucrose pellets preference test is additional reward-based test and a measure of anhedonia. All pellets were prepared by mixing the same weight of flour and water. The white and yellow pellets were supplemented with 0.5 g tasteless food coloring, 1 g of sucrose was randomly added to white and yellow pellets. The weight of every pellet was 20 g. To avoid color interference with the appetite, the double-blind experiments were designed, the preparation of pellets and data statistics were operated by different technicians. Before the formal test, the mice were adapted to the sweet sucrose pellets for 48 hours. Then, after 12 hours of food and water deprivation, mice were provided with sweet and unsweetened pre-weighed pellets for 6 hours. Total weight of every color pellet remaining at the end of 6 hours was recorded. The sucrose pellets preference was calculated according to the formula % preference = [sweet pellets intake/ (sweet + unsweet pellets total intake)] × 100%.

**Tail suspension test (TST)**

The tail suspension is a behavioral despair-based test. Mice were suspended by its tail around 2 cm from the tip at a height of 20 cm. Behavior was recorded for 6 minutes. The duration of immobility in the last 4 minutes was calculated by the Labstate software (ver. 2.0) (YHTSM, Wuhan Yihong Technology Co., Ltd China) [2, 9].

**Forced swimming test (FST)**

The forced swimming is a despair-based behavioral test. Each mouse was trained to swim for 15 minutes on the first day. Next day the mouse was put into a glass cylinder that contained 30 cm deep water (25 ± 1°C) for 6 minutes. The time of immobility was recorded during the last 4 minutes period which followed 2 minutes of habituation [10].

**Open field test**

The open field test evaluates the autonomous activity behavior, exploration behavior and anxiety of experimental animals in a new environment. The mice were placed in the open field box (50 × 50 × 50 cm), the central area covers one-third of the total open field area and behaviors were recorded for 5 minutes by the Labstate (ver. 2.0) system (YHOFM, Wuhan Yihong Technology Co., Ltd China). The parameters used for analysis included the total travel distance and time spent in the central area [11].

**Three**-**chambered sociability test**

Three-chambered sociability test measures the social exploration of mice. The three-chamber device is a Perspex box (60 × 40 cm). The device has two gated walls dividing it into three chambers: empty, central, and [stranger](#/javascript:;). With the gates to both chambers closed, each test mouse was placed in the central chamber for 5 minutes and then removed. A stranger mouse was placed into the transparent plastic cage in the [stranger](#/javascript:;) chamber to avoid direct contact with the test mice, while the cage on the other side was left empty. In the test, the gates open for the test mice to explore the whole chamber, the mice were allowed to roam freely in three chambers for 10 minutes. The movements of mice and time spent in each chamber and in social circles (the area around the strange mice cage 3 cm from the cage is defined as social circle) were recorded by a video-tracking system Labstate (ver. 2.0) (YHSAM, Wuhan Yihong Technology Co., Ltd China). Chamber duration rate (%) = chamber time/total time × 100%, social circles duration rate (%) = social Circles time/total time × 100% [12].

**Pentobarbital induced sleep test**

Pentobarbital induced sleep test is a common method for evaluating sleep in mice. The test was set to take place between 1: 00 pm and 5: 00 pm. Each test mouse was intraperitoneally injected with pentobarbital sodium (50 mg/kg). When mouse is placed in dorsal decubitus position, it turns back to its normal position immediately executing the righting reflex. When the righting reflex disappears for more than 30 seconds the mouse was considered asleep. The sleep latency (the time spent between injection of pentobarbital sodium and disappearance of the righting reflex) and sleep duration (the time spent between the mice asleep and appearance of the righting reflex) were recorded [13].

**Total sleep time calculation**

In order to explore the sleep duration, we conducted a 24 hours video recording, from which the sleep duration was measured manually.

**Morris water maze test**

The Morris water maze test was a spatial learning and memory test. The mice were trained 5 consecutive days daily with four trials, during this period, the mice were trained from different a starting quadrant to locate and escape onto the platform. The platform position was fixed throughout the test. Animals that failed to find the location within 60 seconds were guided to the platform and were allowed to remain on it for 20 seconds. On the sixth day, the platform was removed, and the mice were given 60 seconds to explore, and the time spent in the target quadrant was collected for each mouse [6, 8].

**Rotating rod test**

Rotating rod test assesses the balancing ability as a proxy for motor control by recording time spent on the rotating rod. Each mouse was placed on a rotating bar, which was set to a rotation speed of up to 20 rpm during the test. The time spent on the rotating bar was recorded as the latent period. The latency before falling was recorded using a stop watch, with a maximum of 90 seconds [6, 8, 11].

**Pole test**

Each mouse was paced head-upward on the top of a vertical rough-surfaced pole (diameter 1 cm; height 55 cm). The turn downward from the top of pole (T-turn time) and the descent to the floor (T-LA) time was recorded [6].

**Bite ability test**

The bite ability test was used to evaluate the bite force of mice, which could indirectly reflect the irritability and physical strength of the mice. Mice were given an apple tree branch of the same shape, size and weight between 1:00 am and 3:00 am. The weight of the torn debris was measured.

**Assessment of glymphatic system**

Mice were anaesthetized with a mixture of ketamine (100 mg/kg) and xylazine (10 mg/kg) by intraperitoneal injection. The fluorescence tracers (OA555, FITC-D3) were reconstituted in artificial cerebrospinal fluid (ACSF) at a concentration of 0.5%. Mice were anesthetized and fixed in a stereotaxic frame while the posterior atlanto-occipital membrane was surgically exposed. Using a 30 GA needle, the tracer was infused into the subarachnoid CSF via cisterna magna puncture at a rate of 2μl/min for 5 minutes (10 μl total volume). 30 minutes after the start of infusion, anesthetized animals were transcardially perfusion fixed with 4% paraformaldehyde (PFA). Brain tissue was cut into 50 μm slice and was imaged using Carl Zeiss Axio Scan microscope (Promenade 10, Jena, Germany) [6].

**Pharmacological treatment**

LiCl was intraperitoneally injected at a dose of 45 mg/kg/day; valproic acid (VPA) was intraperitoneally injected at a dose of 120 mg/kg/day in the three weeks of CURD regimen.

**Western blotting**

Using bovine serum albumin (BSA) as the standard, the protein concentration of the sample was determined by the Lowry method. Each sample contained 100 g protein was added into 10% SDS-polyacrylamide gel electrophoresis. After electrophoretic separation and the gels were transferred to polyvinylidene fluoride (PVDF) membranes, the samples were blocked by 5 % skimmed milk powder for 1 hour, and membranes were incubated overnight with the primary antibodies, specific to either SERT at 1:1000 dilution, β-actin at 1:1000 dilution. After washing, specific binding was detected by horseradish peroxidase-conjugated secondary antibodies. Staining was visualized by electrochemiluminescence (ECL) detection reagents and analyzed with an Electrophoresis Gel Imaging Analysis System (MF-ChemiBIS 3.2, DNR Bio-Imaging Systems, Israel). Band density was measured with Window AlphaEaseTM FC 32-bit software [14].

**Immunofluorescence (IF)**

The anaesthetized mice were perfused through the heart with 4% paraformaldehyde (PFA) for 15 minutes. After dissection the brain tissue was cut into 60 μm slices. Brain slices were permeabilized by incubation for 1 hour with donkey serum. Primary antibodies against *c-fos* were used at 1:100 dilution, against NeuN was used at 1:100 dilution. And nuclei were stained with marker 4’, 6’-diamidino-2-phenylindole (DAPI) at 1:1000 dilution. The incubation with the primary antibodies were overnight at 4 °C and then donkey anti-mouse or anti-rabbit Alexa Fluor 488/555 conjugated secondary antibodies were incubated for 2 hours at room temperature. Images were captured using a confocal scanning microscope (DMi8, Leica, Wetzlar, Germany) [15].

**Microdialysis and HPLC-MS analysis**

Mice were anaesthetized by a mixture of ketamine (100 mg/kg) and xylazine (10 mg/kg) by intraperitoneal injection. A guide cannula (CMA 7, CMA Microdialysis, Stockholm, Sweden) was implanted into the right prefrontal cortex (coordinates: anteroposterior 1.75 mm, mediolateral 0.75 mm, dorsoventral 1.5 mm). A microdialysis probe (CMA 7; molecular weight cut-off, 6,000 Da) was inserted through the cannula 24 hours before the start of experiments. Artificial cerebrospinal fluid (ACSF) was perfused through the microdialysis probe at 1 μL/min, and samples were collected 3 hours after probe insertion. The interstitial fluid 5-HT levels were measured immediately [9]. Analyses of serotonin were performed with a high-performance liquid chromatography (HPLC; Agilent 1260 Infinity LC system) tandem with a mass spectrometry (MS) system, Agilent 6420 triple-quad mass spectrometer (Agilent Technologies, CA, USA). A mobile phase composed of acetonitrile (ACN), solvent A, and 0.2% formic acid in water, solvent B, was used. In the HPLC-MS system, 5 μL of the samples was injected into a 120 SB-C18 column (Poroshell, Agilent, 46×100 mm). The experiments were effectuated at 20°C (room temperature) for 5 min with an elution gradient (0-2 min: 0.25 mL/min; 2-5 min: 0.5 mL/min; 0-5 min 70% solvent A, 30% solvent B). Before injections, the column was equilibrated for 15 min. The mass spectrometer parameters were as follows: positive multiple reaction monitoring mode MRM (177.2→160.2; 177.2→132.2), positive electrospray ionisation (ESI), collision energy: 15, 10 L/min heating gas flow, 300°C interface temperature, 3 L/min nebulising gas flow, 300 °C DL temperature, 400°C heat block temperature, and 10 L/min drying gas flow. The detection limit was 1 pmol per injection [16]. The quantification was performed using peak area ratios from calibration standard curves and was normalized to the total protein levels in the samples as determined by the Lowry method.

**Fluorescence activated cell sorter (FACS)**

B6-Tg-Aldh1l1-EGFP mice were used for isolating astrocytes. A cell suspension from the cortex was prepared as previously described [11, 17]. The cortices from three mice were pooled into one sample. Wavelengths for EGFP excitation and emission were 488 and 530/30 nm, respectively. The labeled cells were sorted and collected using the BD FACSAria Cell Sorting System (35 psi sheath pressure, FACSDiva software S/W 2.2.1; BD Biosciences, San Jose, CA) [11, 18]. The purity of sorted astrocytic populations has been ascertained by detecting mRNA of the cell specific marker as described previously [7, 17, 19, 20].

**Clinical Sample Collection and Preparation**

This study was authorized and approved by Medical Ethics Committee of China Medical University (No. [2022]452, No. [2023]58) and registered on Chinese Clinical Trial Registry (registration number: ChiCTR2200061158). Blood samples were collected from 30 healthy subjects, 30 patients with major depressive disorder (MDD) patients and 30 patients with bipolar disorder (BD), diagnosed in the First Hospital of China Medical University. Both manic and depressive symptoms of patients were also estimated by the Clinician-Administered Rating Scale for Mania (CARS-M) [21] and the Hamilton Depression Rating Scale (HDRS) [22], irrespectively. The demographics of health subjects and patients were shown in Supplementary Table 2.

Blood samples were drawn from the median cubital veain and transferred into vacutainer tubes containing ethylene diamine tetracetic acid (EDTA). Samples were centrifuged immediately at 4000×g for 5 min at 4◦C. The obtained plasma samples were stored in the darkness at −80◦C until the measurements.

**Arachidonic Acid (AA) and Prostaglandin E2 (PGE2) Assays**

The collected plasma from the wild type mice and the collected plasmas from health subjects and the diagnosed patients of BD and MDD were used to measure the levels of AA and PGE2. Different concentrations of AA standard solution were prepared by multiple dilution method, with concentrations of 100 ng/ml,50 ng/ml, 25 ng/ml, 12.5 ng/ml, 6.25 ng/ml and 3.13 ng/ml. Similarly, standard solution concentrations of PGE2 were 2000 pg/ml, 1000 pg/ml, 500 pg/ml, 250 pg/ml, 125 pg/ml, 62.5 pg/ml, 31.25 pg/ml. 50 μL of sample or standard solution and 50 μL of biotinylated antibody working solution were added to each hole on the enzyme plate. In addition, we also set up blank control holes, in which 50 μL of standard diluent and 50 μL of biotinylated antibody working solution were added. The enzyme plates were incubated at 37 °C for 45 minutes, dry the liquid in the hole and wash it three times. 100 μL of horseradish peroxidase (HRP) conjugate working solution was added to each hole and the holes were washed 5 times after incubation at 37 °C for 45 minutes. 90 μL of 3,3',5,5'-tetramethylbenzidine (TMB) substrate solution was added into each hole, incubated at 37 °C away from light for 15 minutes, 50 μL of terminating solution was added to terminate the reaction, and then the optical density was detected by multi-function enzyme labeling instrument immediately, then the standard curves were made by the measured optical densities of the standard solutions to calculate the concentrations of AA and PGE2 in the samples. Results were finally normalized by the protein content.

**Statistics and Reproducibility**

Power Analysis and Sample Size (PASS) 2020 software (NCSS, LLC, Utah, USA) was used to estimate the suitable sample number in clinical and animal experiments. For statistical analysis one-way analysis of variance (ANOVA) followed by a Tukey’s or Dunnett’s post hoc multiple comparison test for unequal replications were used in GraphPad Prism 5 software (GraphPad Software Inc., La Jolla, CA) and SPSS 24 software (International Business Machines Corp., NY, USA). One-way ANOVA for comparisons including more than two groups; unpaired two-tailed t-test for two group comparisons. All statistical data in the text are presented as the mean ± SD, the value of significance was set at p < 0.05. For correlation analysis, Spearman correlation analysis on the data, analysis and visualization was performed using R 3.6.3 and Empower (R) software (X&Y Solutions, Inc., MA, USA), and operated R package ggplot2 to do visualization.

**References**

1. Tran HQ, Shin EJ, Saito K, Tran TV, Phan DH, Sharma N, et al. Indoleamine-2,3-dioxygenase-1 is a molecular target for the protective activity of mood stabilizers against mania-like behavior induced by d-amphetamine. Food Chem Toxicol. 2020;136:110986.

2. Tang M, He T, Sun X, Meng QY, Diao Y, Lei JY, et al. Subregion-specific decreases in hippocampal serotonin transporter protein expression and function associated with endophenotypes of depression. Hippocampus. 2014;24:493-501.

3. Freitas AE, Egea J, Buendia I, Gómez-Rangel V, Parada E, Navarro E, et al. Agmatine, by Improving Neuroplasticity Markers and Inducing Nrf2, Prevents Corticosterone-Induced Depressive-Like Behavior in Mice. Mol Neurobiol. 2016;53:3030-45.

4. Wolfe AM, Kennedy LH, Na JJ, Nemzek-Hamlin JA. Efficacy of Tramadol as a Sole Analgesic for Postoperative Pain in Male and Female Mice. J Am Assoc Lab Anim Sci. 2015;54:411-9.

5. Xia M, Liang S, Li S, Ji M, Chen B, Zhang M, et al. Iatrogenic Iron Promotes Neurodegeneration and Activates Self-Protection of Neural Cells against Exogenous Iron Attacks. Function (Oxf). 2021;2:zqab003.

6. Liang S, Lu Y, Li Z, Li S, Chen B, Zhang M, et al. Iron Aggravates the Depressive Phenotype of Stressed Mice by Compromising the Glymphatic System. Neurosci Bull. 2020;36:1542-6.

7. Li B, Dong L, Wang B, Cai L, Jiang N, Peng L. Cell type-specific gene expression and editing responses to chronic fluoxetine treatment in the in vivo mouse brain and their relevance for stress-induced anhedonia. Neurochem Res. 2012;37:2480-95.

8. Xia M, Yang L, Sun G, Qi S, Li B. Mechanism of depression as a risk factor in the development of Alzheimer's disease: the function of AQP4 and the glymphatic system. Psychopharmacology (Berl). 2017;234:365-79.

9. Xia M, Li Z, Li S, Liang S, Li X, Chen B, et al. Sleep Deprivation Selectively Down-Regulates Astrocytic 5-HT(2B) Receptors and Triggers Depressive-Like Behaviors via Stimulating P2X(7) Receptors in Mice. Neurosci Bull. 2020;36:1259-70.

10. Florensa-Zanuy E, Garro-Martínez E, Adell A, Castro E, Díaz Á, Pazos Á, et al. Cannabidiol antidepressant-like effect in the lipopolysaccharide model in mice: Modulation of inflammatory pathways. Biochem Pharmacol. 2021;185:114433.

11. Li X, Liang S, Li Z, Li S, Xia M, Verkhratsky A, et al. Leptin Increases Expression of 5-HT(2B) Receptors in Astrocytes Thus Enhancing Action of Fluoxetine on the Depressive Behavior Induced by Sleep Deprivation. Front Psychiatry. 2018;9:734.

12. Li Z, Lu Y, Liang S, Li S, Chen B, Zhang M, et al. Fluoxetine improves behavioural deficits induced by chronic alcohol treatment by alleviating RNA editing of 5-HT(2C) receptors. Neurochem Int. 2020;134:104689.

13. Kim JW, Kim CS, Hu Z, Han JY, Kim SK, Yoo SK, et al. Enhancement of pentobarbital-induced sleep by apigenin through chloride ion channel activation. Arch Pharm Res. 2012;35:367-73.

14. Chen B, Zhang M, Ji M, Zhang D, Chen B, Gong W, et al. The neuroprotective mechanism of lithium after ischaemic stroke. Commun Biol. 2022;5:105.

15. Yue T, Li B, Gu L, Huang J, Verkhratsky A, Peng L. Ammonium induced dysfunction of 5-HT(2B) receptor in astrocytes. Neurochem Int. 2019;129:104479.

16. Ren J, Li X, Sun G, Li S, Liang S, Li Z, et al. Protective effect of leptin-mediated caveolin-1 expression on neurons after spinal cord injury. Cell Calcium. 2018;76:122-8.

17. Guan W, Xia M, Ji M, Chen B, Li S, Zhang M, et al. Iron induces two distinct Ca(2+) signalling cascades in astrocytes. Commun Biol. 2021;4:525.

18. Ji M, Gong W, Wang S, Zhang D, Chen B, Li X, et al. Leptin Attenuates Fear Memory by Inhibiting Astrocytic NLRP3 Inflammasome in Post-traumatic Stress Disorder Model. Neurochem Res. 2022.

19. Fu H, Li B, Hertz L, Peng L. Contributions in astrocytes of SMIT1/2 and HMIT to myo-inositol uptake at different concentrations and pH. Neurochem Int. 2012;61:187-94.

20. Zhao X, Li Z, Liang S, Li S, Ren J, Li B, et al. Different epidermal growth factor receptor signaling pathways in neurons and astrocytes activated by extracellular matrix after spinal cord injury. Neurochem Int. 2019;129:104500.

21. Altman EG, Hedeker DR, Janicak PG, Peterson JL, Davis JM. The Clinician-Administered Rating Scale for Mania (CARS-M): development, reliability, and validity. Biol Psychiatry. 1994;36:124-34.

22. Hamilton M. Development of a rating scale for primary depressive illness. Br J Soc Clin Psychol. 1967;6:278-96.
